# Enhanced Monocyte Response and Decreased Central Memory T Cells in Children with Invasive *Staphylococcus aureus* Infections

**DOI:** 10.1371/journal.pone.0005446

**Published:** 2009-05-08

**Authors:** Monica I. Ardura, Romain Banchereau, Asuncion Mejias, Tiziana Di Pucchio, Casey Glaser, Florence Allantaz, Virginia Pascual, Jacques Banchereau, Damien Chaussabel, Octavio Ramilo

**Affiliations:** 1 Division of Pediatric Infectious Disease, Department of Pediatrics, University of Texas Southwestern Medical Center, Dallas, Texas, United States of America; 2 Children's Medical Center, Dallas, Texas, United States of America; 3 Baylor NIAID Cooperative Center for Translational Research on Human Immunology and Biodefense, Dallas, Texas, United States of America; 4 Baylor Institute for Immunology Research and Baylor Research Institute, Dallas, Texas, United States of America; Columbia University, United States of America

## Abstract

*Staphylococcus aureus* has emerged as a significant pathogen causing severe invasive disease in otherwise healthy people. Despite considerable advances in understanding the epidemiology, resistance mechanisms, and virulence factors produced by the bacteria, there is limited knowledge of the *in vivo* host immune response to acute, invasive *S. aureus* infections. Herein, we report that peripheral blood mononuclear cells from patients with severe *S. aureus* infections demonstrate a distinctive and robust gene expression profile which is validated in a distinct group of patients and on a different microarray platform. Application of a systems-wide modular analysis framework reveals significant over-expression of innate immunity genes and under-expression of genes related to adaptive immunity. Simultaneous flow cytometry analyses demonstrated marked alterations in immune cell numbers, with decreased central memory CD4 and CD8 T cells and increased numbers of monocytes. CD14+ monocyte numbers significantly correlated with the gene expression levels of genes related to the innate immune response. These results demonstrate the value of applying a systems biology approach that reveals the significant alterations in the components of circulating blood lymphocytes and monocytes in invasive *S. aureus* infections.

## Introduction

*Staphylococcus aureus* has emerged as a leading cause of both community-associated and nosocomial invasive bacterial infections in the United States, causing a wide spectrum of clinical illnesses ranging from superficial soft tissue infections to severe, invasive disease leading to considerable morbidity and mortality in otherwise healthy children and adults [1]–[5]. As such, the US Centers for Disease Control and Prevention has identified *S. aureus* as a major public health problem in a recent population-based surveillance study [6]. The increasing magnitude of *S. aureus* disease burden, changes in bacterial susceptibility, and continued poor patient outcomes with available antimicrobial therapies clearly demonstrate a need to improve our understanding of its pathogenesis in order to develop more effective therapeutic strategies. To date, most efforts have been focused on advancing our knowledge of the pathogen and its remarkable repertoire of virulence factors [7]. To complement this progress, it is critical that we also gain new insight into the host immune response to *S. aureus* infection in a clinically relevant context.

Genome-wide analyses of blood leukocytes provide a global and comprehensive assessment of the complexity of the immune network and response to disease [8]. Previous work has demonstrated the applicability of blood leukocyte microarray data into clinical relevance, allowing for biomarker discovery leading to improved diagnostic and prognostic indicators and innovative therapeutics in autoimmune diseases [9]–[11] and cancer [12]–[15]. With advances in gene expression profiling and application of microarray technology to the field of infectious diseases, we can now more clearly define the host's immune response to a pathogen and identify a unique biosignature that is not limited by traditional diagnostic or microbiologic techniques [16]–[18]. Peripheral blood mononuclear cells (PBMCs) provide an accessible source of molecular phenotypic information as they traffic within the systemic circulation, to and from sites of infection and inflammation. When a pathogen infects the host, specific pattern-recognition receptors expressed on leukocytes recognize unique pathogen-associated molecular patterns displayed on microbes and initiate the host's innate and pro-inflammatory response to the infection while simultaneously activating the adaptive immune response [19], [20]. The microbe-induced changes in host cells demonstrate significant and stereotypical changes at the gene expression level that are unique to the pathogen and can be objectively measured [21], [22]. Genome-wide arrays have been used to advance our understanding of the human host response to febrile illnesses such as malaria [17], acute dengue hemorrhagic fever [17], HIV [23], and Kawasaki syndrome [17]. Our previous work has demonstrated that PBMCs of children with different acute infections carry distinct gene expression patterns that allow discrimination between viral and bacterial pathogens and even among subtypes of bacteria [24]. Despite different bacterial strains and diverse clinical manifestations, we hypothesized that gene expression analysis of PBMCs from children with acute invasive *S. aureus* infections would demonstrate a characteristic and unique host immune response and provide a novel and broad insight into the pathogenesis of these infections. The present study was designed to obtain a comprehensive characterization of the host immune response to invasive *S. aureus* infections in children using a combination of gene expression and flow cytometry analyses.

## Results

### Patient characteristics

Over a period of 4 years, samples from 53 previously healthy patients hospitalized with invasive *S. aureus* infections and 24 healthy control subjects were analyzed. Patients were chosen representing the clinical spectrum of acute severe *S. aureus* disease including bacteremia, osteomyelitis, suppurative arthritis, pyomyositis, and pneumonia with empyema. Patients with a diagnosis of staphylococcal toxic shock syndrome or polymicrobial infections were excluded. Patient demographic data, clinical characteristics, analysis group, and microarray platform are summarized in **Table 1**. There were no statistical differences between the *S. aureus*-infected children and their respective healthy controls with regards to age, sex, or race in the training and test sets (**Table 2**). Patients were enrolled only after a bacteriologic diagnosis was established; the median time from patient hospitalization to procurement of study blood sample was 4 days [IQ range 3–8 days]. Viral direct fluorescent antibody testing or culture of the nasopharynx was obtained on 68 subjects (88%, 46 patients, 22 controls) and did not reveal the presence of a concomitant viral infection.

**Table 1:** Subject characteristics.

SUBJECT #	AGE (yrs)	RACE	SEX	CONDITION	PATHOGEN	ANALYSIS	PLATFORM
3N	6	H	M	Control	Healthy	Training/Test	A
5	10	H	M	Osteomyelitis	MSSA	Training	A
7N	1.6	B	F	Control	Healthy	Training/Test	A
8N	10	B	M	Control	Healthy	Training/Test	A
11N	4	B	F	Control	Healthy	Training/Test	A
23N	7	H	F	Control	Healthy	Training/Test	A
24	3	B	M	Bacteremia, Osteomyelitis, Myositis	MRSA	Test	A
24N	3	W	M	Control	Healthy	Training/Test	A
30	15	B	M	Bacteremia	MRSA	Test	A
40	13	W	M	Bacteremia, Osteomyelitis	MSSA	Training	A
43	7	B	M	Bacteremia, Osteomyelitis, SArthritis, Pyomyositis, Emboli	MRSA	Test	A
62	2	W	M	Osteomyelitis, Pyomyositis	MRSA	Training	A
66	0.25	B	F	Pneumonia	MRSA	Test	A
67	7	W	F	Bacteremia, Osteomyelitis	MRSA	Training	A
88	0.92	H	M	Bacteremia, Osteomyelitis, Pneumonia, Emboli	MRSA	Test	A
90	0.67	B	M	Bacteremia, SArthritis	MSSA	Test	A
109	0.67	H	F	Bacteremia, SST Abscess	MRSA	Training	A
150	9	B	F	Bacteremia, Osteomyelitis, SArthritis, Myositis	MRSA	Test	A
179	12	W	M	Bacteremia, Endocarditis, Emboli	MSSA	Training	A
205	7	H	M	Bacteremia, Pneumonia, SST Abscess	MRSA	Test	A
208	10	W	F	Bacteremia, Osteomyelitis, CNS abscess, Pneumonia, Emboli	MRSA	Test	A
216	10	H	F	Bacteremia, Osteomyelitis	MRSA	Training	A
220	11	H	M	Bacteremia, Osteomyelitis	MSSA	Test	A
221	6	B	F	Bacteremia, Osteomyelitis	MRSA	Training	A
224	10	W	M	Bacteremia, Osteomyelitis	MSSA	Test	A
230	20	B	M	Bacteremia, Endocarditis, SST Abscess	MRSA	Test	A
241	0.92	B	F	Bacteremia, Osteomyelitis, Pneumonia	MRSA	Training	A
242	1.2	B	M	Bacteremia, Osteomyelitis, Pyomyositis	MRSA	Test	A
258	8	W	F	Bacteremia, Osteomyelitis, Cellulitis	MSSA	Training	A
262	13	H	M	Bacteremia, SST Abscess	MRSA	Training	A
264	13	B	M	Bacteremia, Osteomyelitis, SArthritis, SST Abscess, Myositis, Emboli	MSSA	Test	A
271	13	B	M	Osteomyelitis, pyomyositis	MSSA	Training	A
294	12	B	F	Control	Healthy	Training/Test	A
301	8	W	M	Control	Healthy	Training/Test	A
303	6	W	F	Control	Healthy	Training/Test	A
304	6	W	M	Control	Healthy	Training/Test	A
305	4	H	F	Bacteremia, Osteomyelitis, SArthritis	MSSA	Training	A
308	12	B	F	Bacteremia, Pneumonia, Pyomyositis, SST Ab	MSSA	Training	A
328	0.38	B	F	Bacteremia, Osteomyelitis, Pneumonia, SST Abscess	MSSA	Test	A
329	0.58	O	F	Bacteremia, Lymphadenitis	MRSA	Training	A
330	11	B	F	Bacteremia, Pneumonia	MRSA	Test	A
354	2	H	F	Bacteremia, Osteomyelitis	MRSA	Training	A
366	11	B	F	Osteomyelitis, SST Abscess	MRSA	Test	A
369	14	B	M	Bacteremia, SArthritis, SST Abscess, Emboli	MRSA	Test	A
372	14	W	M	Bacteremia, Osteomyelitis, Myositis	MRSA	Test	A
412	1.75	W	M	Bacteremia, SArthritis	MSSA	Test	A
418	8	H	F	Osteomyelitis	MSSA	Training	A
423	8	W	M	Bacteremia, Osteomyelitis, Pyomyositis	MSSA	Training	A
434	5	W	M	Osteomyelitis, Pyomyositis	MSSA	Test	A
440	0.5	B	M	Bacteremia, Osteomyelitis, Pneumonia, Emboli	MRSA	Training	A
450	0.83	B	M	Bacteremia, Osteomyelitis, SArthritis, Pyomyositis	MSSA	Test	A
451	4	H	M	Bacteremia, Osteomyelitis, SArthritis, Pyomyositis, Emboli	MRSA	Training	A
894	8	W	F	Osteomyelitis, SST Abscess	MSSA	Validation, FACS	I
903	13	W	F	Control	Healthy	Validation, FACS	I
904	16	W	F	Control	Healthy	FACS	I
905	16	W	F	Control	Healthy	FACS	I
907	1.3	H	F	Bacteremia	MRSA	FACS	I
908	8	B	M	Bacteremia, Pleural effusion, Pyomyositis, SST Abscess, Emboli	MSSA	FACS	I
909	6	H	F	Bacteremia, Osteomyelitis, SArthritis, Pneumonia	MRSA	Validation, FACS	I
910	5	B	M	Osteomyelitis, SArthritis	MSSA	Validation, FACS	I
922	3	H	F	Control	Healthy	FACS	I
926	3	H	M	Control	Healthy	FACS	I
927	2	H	M	Control	Healthy	Validation, FACS	I
929	11	O	F	Control	Healthy	Validation, FACS	I
930	8	O	M	Control	Healthy	Validation	I
931	12	W	F	Control	Healthy	FACS	I
933	9	W	M	Control	Healthy	Validation, FACS	I
935	9	W	F	Control	Healthy	Validation, FACS	I
939	8	W	M	Control	Healthy	Validation, FACS	I
940	7	W	M	Control	Healthy	Validation, FACS	I
941	9	W	F	Control	Healthy	Validation, FACS	I
943	9	W	M	Osteomyelitis, SArthritis, Pyomyositis	MRSA	Validation, FACS	I
944	6	B	F	Bacteremia, Osteomyelitis, SArthritis, Pyomyositis	MRSA	Validation, FACS	I
949	11	W	M	Bacteremia, Osteomyelitis, SArthritis	MSSA	Validation	I
952	15	B	M	Bacteremia, Osteomyelitis, Pneumonia	MRSA	Validation, FACS	I
954	13	W	M	Bacteremia, Osteomyelitis, Pyomyositis	MSSA	Validation, FACS	I
960	3	H	M	Pneumonia	MRSA	Validation, FACS	I

H = Hispanic, W = White, B = Black, O = Other; M = Male, F = Female; SST Ab = Skin/soft tissue abscess, SArthritis = Suppurative arthritis, CNS = central nervous system; MSSA = methicillin-susceptible *Staphylococcus aureus*, MRSA = methicillin-resistant *Staphylococcus aureu*s; Platform: A = Affymetrix U133 A&B I =  Illumina Sentrix Hu6 BeadChips.

**Table 2:** Demographic and Laboratory Characteristics of Patients and Controls in Training and Test Sets.

*Parameter*	TRAINING SET			TEST SET		
	*Patients*	*Controls*	*p* a	*Patients*	*Controls*	*p* a
**Age (years)**	7.5 [2–11]	6 [3.5–9]	0.86	7 [1–12]	6 [3.5–9]	0.93
**Race**	5B,8H,6W,1O	4B,2H,4W	1	14B,3H,5W	4B,2H,4W	0.25
**Gender**	9M:11F	5M:5F	0.57	16M:6F	5M:5F	0.45
**WBC (thousand/mm^3^)**	8.9 [7.5–17.4]	7.2 [5.2–8.2]	0.03	11 [7.7–16]	7.2 [5.2–8.2]	0.009
**Neutrophils (%)**	60 [48–73]	43 [27–50]	0.007	61 [40–66]	43 [27–50]	0.022
**Lymphocytes (%)**	24 [13–38]	47 [39–55]	0.002	24 [12.7–47]	47 [39–55]	0.038
**Monocytes (%)**	8 [7–11]	7 [6–10]	0.37	9 [6.3–12.5]	7 [6–10]	0.38
**Hematocrit (%)**	31.6 [29.8–36]	37.7 [34.9–40]	0.007	30 [27–33.9]	37.7 [34.9–40]	0.002
**Platelets (thousand/mm^3^)**	382 [297–459]	310 [264–329]	0.07	353 [273–445]	310 [264–329]	0.373
**CRP (mg/dL)**	6.65 [2.1–16.5]	0.4 [0.4–0.7]	<0.001	7.4 [2.3–16.9]	0.4 [0.4–0.7]	0.001

Median values [25–75% range]; B = Black, H = Hispanic, W = White, O = Other; M = Male, F = Female; CRP = C-reactive protein.

a p values calculated within each individual subject set and respective, matched controls (Mann-Whitney).

Patients with culture-proven invasive *S. aureus* infections were divided into 3 groups for analysis: training, test, and validation sets. The training set of subjects composed of 20 children with invasive *S. aureus* infections (median age 7.5 years; 11 methicillin-resistant *S. aureus*, MRSA, and 9 methicillin-susceptible *S. aureus*, MSSA) and 10 healthy controls (median age 6 years) matched for age, sex, and race, were initially analyzed to identify the gene expression profile in PBMCs from *S. aureus*-infected patients. As expected, there were statistical differences in laboratory parameters with higher total peripheral white blood cell count and percent neutrophil count, but lower percent lymphocyte count and hematocrit values in patients with *S. aureus* infections compared with healthy controls (**Table 2**). The test set of subjects included an independent group of 22 patients with *S. aureus* infection (median age 7 years; 8 MSSA, 14 MRSA) and 10 healthy controls (median age 6 years) and was used to validate the gene expression profile in PBMCs from *S. aureus*-infected patients. As in the training set, there were differences in laboratory values between patients with *S. aureus* infection and healthy controls (**Table 2**).

A third independent group of 25 subjects was included to validate our initial findings using (1) a second microarray platform (Illumina) and (2) flow cytometry. This validation set was comprised of 11 patients with *S. aureus* infection (median age 8 years; 5 MSSA, 6 MRSA) and 13 healthy controls (median age 9 years). PBMCs from 23 subjects were evaluated by flow cytometry to determine the relative abundance of different immune cell populations. Simultaneous flow cytometry evaluation and gene expression analysis was conducted with the same PBMC samples in 18 (9 with *S. aureus* infection and 9 healthy controls, matched for age, sex, race) of these 23 subjects. There were no statistical differences in extent of disease severity, antimicrobial therapy, or demographic and laboratory data between the training, test, and validation sets (**Table 3**).

**Table 3:** Demographic and Laboratory Characteristics of Patients in Training, Test, and Validation Sets.

S. aureus *patients*	Training Set	Test Set	Validation Set	p-value^a^
**Age (years)**	7.5 [2–11]	7 [1–12]	8 [5–11]	0.82
**Race**	5B,8H,6W,1O	14B,3H,5W	4B,3H,4W	0.08
**Gender**	9M:11F	16M:6F	7M:4F	1
**WBC (thousand/mm^3^)**	8.9 [7.5–17.4]	11 [7.7–16]	8.5 [5.7–15.5]	0.52
**Neutrophils (%)**	60 [48–73]	61 [37–66]	55 [45–71]	0.73
**Lymphocytes (%)**	24 [13–38.3]	24 [12.7–47.5]	29 [19.5–44.5]	0.70
**Monocytes (%)**	8 [7–11]	9 [6.3–12.5]	7 [3.5–11.5]	0.58
**Hematocrit (%)**	31.6 [29.8–36.2]	30 [27.2–33.9]	32 [29–34.1]	0.08
**Platelets (thousand/mm^3^)**	382 [297–459]	353 [273–445]	364 [313–473]	0.61
**ESR (mm/hr)**	70 [34–98]	46 [36–72]	72 [46–87]	0.28
**CRP (mg/dL)**	6.7 [2.1–16.5]	7.4 [2.4–16.9]	7.7 [3.7–21.3]	0.69

Median values [25–75% range]; B = Black, H = Hispanic, W = White, O = Other; M = Male, F = Female; CRP = C-reactive protein.

a p values calculated within each individual subject set and respective to matched controls (Kruskal-Wallis).

### Gene expression signature in patients with invasive S. aureus infections

**Figure 1:**
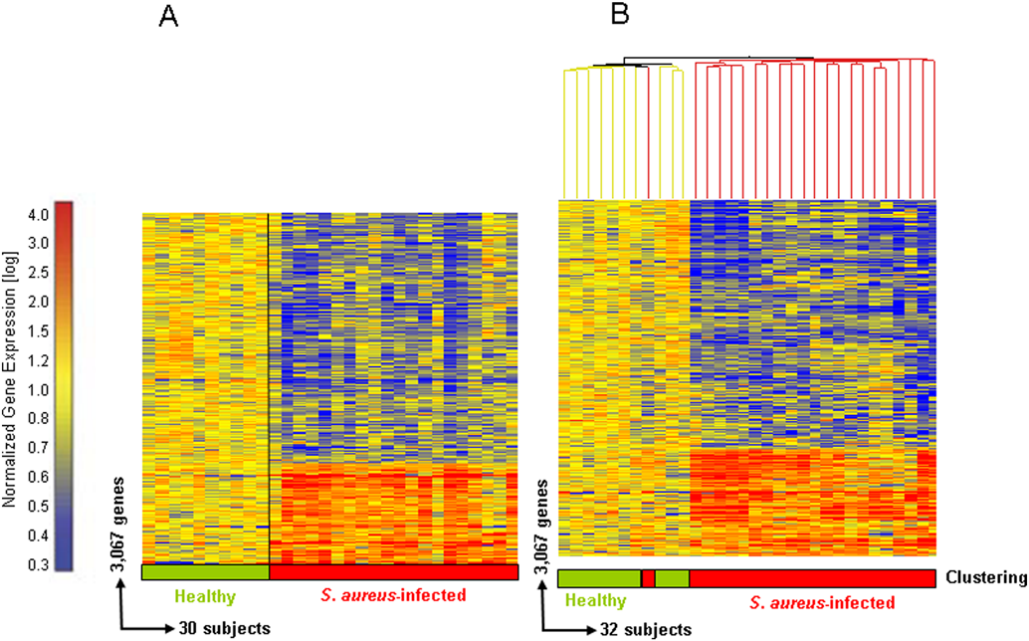
Gene expression biosignature in PBMCs from *S. aureus* patients and healthy controls. (a) Statistical group comparisons between 10 healthy subjects and 20 patients with acute *S.aureus* infections yielded 3,067 genes expressed at statistically different levels (Mann-Whitney p<0.01 and 1.25 fold change) between the two groups. Significant genes were organized by hierarchical clustering to reveal differential expression, each row representing a single gene and each column an individual subject. Transformed expression levels are indicated by color scale: red representing relatively high and blue relatively low gene expression compared to the median expression for each gene across all patients compared to healthy controls. (b) The same 3,067 gene list was used to perform a condition tree on 22 new subjects with *S. aureus* infections and correctly grouped 21 of the 22 patients based solely on gene expression.

Statistical group comparison (Mann-Whitney, p<0.01) was applied to the class comparisons on the quality control (QC) genes present in the training set revealing 3,168 genes differentially expressed between *S. aureus*-infected patients and healthy controls. These genes were then filtered to include those transcripts with a 1.25-fold or greater change in expression level relative to the healthy control group, for a total of 3,067 genes. A hierarchical clustering algorithm was applied to these 3,067 genes in order to visualize the transcriptional pattern (**Figure 1a**). For purposes of validation, the 3,067 gene list comprising the gene expression profile in PBMC of *S. aureus*-infected patients was then evaluated in an independent test set of 22 new patients. The samples were organized into a condition tree utilizing the 3,067 genes and correctly classified 31 of 32 samples as either healthy or *S. aureus* infection based on the gene expression patterns (**Figure 1b**).

Genes represented in the expression profile in PBMCs from *S. aureus*-infected patients were then ranked according to differences in both fold-change and significance in gene expression levels (p<0.05) compared with healthy controls. The top 50 genes that were significantly over-expressed in patients with *S. aureus* infection versus healthy controls are shown in **Table 4**. Over-expressed genes included those with microbicidal functions (lactotransferrin, alpha-defensins 1 and 4, bactericidal/permeability-increasing protein), involved in coagulation (thrombomodulin), hemoglobin synthesis (hemoglobin D and G), and pro-inflammatory and immune-related genes related to pathways of cellular growth, proliferation, and apoptosis (ADM, ARG1, CLU, EGR1, IL8, HBEGF, ITGA2B, MMP9) and involved in cell to cell signaling such as CEACAM6 and CEACAM8.

**Table 4:** Top 50 over-expressed genes in invasive *S. aureus* infections.

Rank	Common Name	Gene Bank Symbol	Description	Fold Change	Significance
1	LTF	NM_002343	Lactotransferrin	46.266	0.000029
2	CEACAM8	M33326	Carcinoembryonic antigen-related cell adhesion molecule 8	31.985	0.000052
3		AW337833	Transcribed locus	32.904	0.000029
4	IL8	NM_000584	Interleukin 8	21.581	0.000075
5	HBG1	NM_000559	Hemoglobin, gamma A	21.543	0.000705
6	DEFA4	NM_001925	Defensin, alpha 4, corticostatin	20.559	0.000075
7	DEFA1	NM_004084	Defensin, alpha 1, myeloid-related sequence	17.011	0.000043
8	HBG2	NM_000184	Hemoglobin, gamma G	15.804	0.001540
9	LCN2	NM_005564	Lipocalin 2 (oncogene 24p3)	13.696	0.000090
10	HBD	NM_000519	Hemoglobin, delta	13.327	0.000432
11	ARG1	NM_000045	Arginase, liver	14.386	0.000366
12	BPI	NM_001725	Bactericidal/permeability-increasing protein	12.791	0.000129
13	HPR	NM_020995	Haptoglobin-related protein	12.218	0.000155
14	S100P	NM_005980	S100 calcium binding protein P	11.571	0.000052
15	THBD	NM_000361	Thrombomodulin	9.005	0.000309
16	ADM	NM_001124	Adrenomedullin	8.670	0.000020
17	CA1	NM_001738	Carbonic anhydrase I	8.627	0.000432
18	ERAF	NM_016633	Erythroid associated factor	7.890	0.000155
19	MSCP	H69701	Mitochondrial solute carrier protein	7.635	0.000043
20	MYL9	NM_006097	Myosin, light polypeptide 9, regulatory	7.509	0.000510
21	MMP9	NM_004994	Matrix metalloproteinase 9 (gelatinase B, 92 kDa gelatinase, 92 kDa type IV collagenase)	7.023	0.00580
22	RETN	NM_020415	Resistin	6.900	0.000108
23	ANXA3	M63310	Annexin A3	6.465	0.000705
24	CEACAM6	M18728		5.918	0.002400
25	MGAM	NM_004668	Maltase-glucoamylase (alpha-glucosidase)	5.823	0.000432
26	PBEF1	AI681868	Pre-B-cell colony enhancing factor 1	5.630	0.001540
27	CYP4F3	NM_000896	Cytochrome P450, family 4, subfamily F, polypeptide 3	5.451	0.000432
28	PBEF	AA873350	Pre-B-cell colony enhancing factor 1	5.407	0.000600
29	ITGA2B	AF098114	Integrin, alpha 2b (platelet glycoprotein IIb of IIb/IIIa complex, antigen CD41B)	5.238	0.000827
30	HP	NM_005143	Haptoglobin	5.138	0.000035
31	SNCA	BG260394	Synuclein, alpha (non A4 component of amyloid precursor)	4.891	0.000016
32	FCGR3A	J04162	Fc fragment of IgG, low affinity IIIb, receptor for (CD16)	4.877	0.000029
33	EGR1	AI459194	Early growth response 1	4.422	0.003200
34	LOC199675	BF433657	Hypothetical protein LOC199675	4.420	0.000309
35	SOCS3	AI244908	qj98g11.x1 NCI_CGAP_Kid3 Homo sapiens cDNA clone IMAGE:1867556 3′, mRNA sequence.	4.312	0.000013
36	EREG	NM_001432	Epiregulin	4.192	0.002070
37	DTR	M60278	Heparin-binding EGF-like growth factor	4.087	0.002400
38	TCN1	NM_001062	Transcobalamin I (vitamin B12 binding protein, R binder family)	3.975	0.007280
39	MS4A3	L35848	Membrane-spanning 4-domains, subfamily A, member 3 (hematopoietic cell-specific)	3.966	0.002780
40	GLUL	NM_002065	Glutamate-ammonia ligase (glutamine synthase)	3.932	0.000366
41		N63920	FP15737	3.883	0.000062
42	FLJ31978	AI041543	Hypothetical protein FLJ31978	3.879	0.000261
43	CLU	M25915	Clusterin (complement lysis inhibitor, SP-40,40, sulfated glycoprotein 2, testosterone-repressed prostate message 2, apolipoprotein J)	3.733	0.000827
44	MAD	AW071793	MAX dimerization protein 1	3.715	0.000016
45	H1F0	BC000145	H1 histone family, member 0	3.713	0.000043
46	FCGR1A	X14355	Fc fragment of IgG, high affinity Ia, receptor for (CD64)	3.712	0.000029
47	SOCS3	BG035761	Suppressor of cytokine signaling 3	3.708	0.000013
48	MS4A4A	NM_024021	Membrane-spanning 4-domains, subfamily A, member 4	3.648	0.001790
49	PBEF	NM_005746	Pre-B-cell colony enhancing factor 1	3.611	0.000155
50	ORF1-FL49	AL522667	Putative nuclear protein ORF1-FL49	3.594	0.000155

### Module-level analysis reveals over-expression of innate and under-expression of adaptive immune response genes

**Figure 2:**
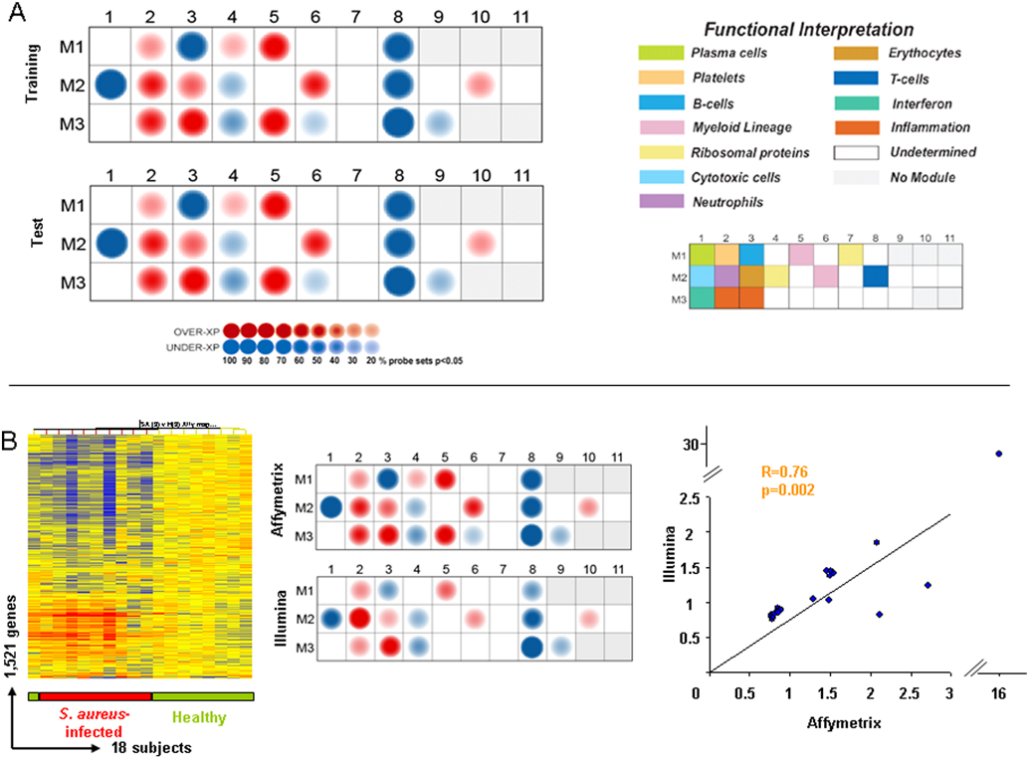
Module analysis identifies a specific gene expression profile in the PBMCs of *S. aureu*s-infected patients. (a) Gene expression levels were compared between patients with *S. aureus* infections and healthy controls on a module-by-module basis. Colored spots represent the percentage of significantly over-expressed (red) or under-expressed (blue) transcripts (p<0.05, Mann Whitney) within a module in patients with *S. aureus* infections; spot intensity represents the magnitude of the gene expression change, blank modules demonstrate no significant differences between groups (p>0.05). Information is displayed on a grid, with the coordinates corresponding to one of 28 modules with the key (upper right) representing the functional interpretation of modules. Nineteen modules are shown to be significantly different between healthy subjects and patients with *S. aureus* infection in the training set (a, upper module map). The same gene list applied to the independent test set of patients reveals the same modular map (a, lower module map). (b) Transcript sequences (RefSeq) were used to map the corresponding 3,067 significant gene probe sets comprising the gene expression profile of PBMC in patients with *S. aureus* infection on the Affymetrix platform to their corresponding 1,521 gene probes on the Illumina platform (b, far left panel) and tested in new *S. aureus* patients (n = 9) and controls (n = 9). Module analysis (b, middle panel) and correlation analyses (Spearman) between the average gene expression levels per module in each platform (b, right panel) were performed.

To better characterize the biological significance of the gene expression profiles seen in the PBMCs of patients with *S. aureus* infections, gene expression levels between patients and healthy controls were mapped using a modular analysis framework that we recently described [25]. A key to the functional interpretation of each transcriptional module is detailed in **Table S1**. Gene expression levels were compared between patients and healthy controls on a module-by-module basis. The percentage of genes with a significant change (Mann-Whitney p<0.05) within each module are graphically displayed on a module map, with over-expressed genes represented in red and under-expressed genes in blue (**Figure 2a**). Following the approach described previously with the class comparisons analysis, module analysis was applied initially to the training set of patients. Patients with *S. aureus* infection demonstrated significant over-expression of genes in modules related to innate immunity including myeloid (M1.5, M2.6), neutrophil (M2.2), and inflammation (M3.2, M3.3) modules and under-expression of genes regulating adaptive immunity such as B cell module M1.3, cytotoxic cell module M2.1, and T cell specific module M2.8. The levels of expression of each transcriptional gene profile significantly changed (Mann Whitney p<0.05) in the PBMCs of patients with *S. aureus* infection versus controls are detailed in **Figure S1**.

Significantly over-expressed genes included those in: M1.5 and M2.6 (“Myeloid”), genes related to cells of the myeloid lineage which are involved in bacterial pathogen recognition such as TLR 2 and CD14, IL10 signaling (CD32, BLVRA) and leukocyte extravasation signaling (CTNNA1, NCF2, PECAM1, ITGB2), also in M2.6 genes related to the inflammatory response (calgranulin B, NFKB inhibitor, TNF superfamily members, and metalloproteinase inhibitors); in M2.2 (“Neutrophils”) genes encoding innate molecules including LTF, DEFA 1 and 4, BPI, CEACAM 8; in modules M3.2 and M3.3 (“Inflammation I and II”) genes involved in inflammatory and endothelial cell processes. Significant genes found over-expressed in M3.2 (“Inflammation II”) included those involved in coagulation (THBD), endovascular inflammation, TLR signaling (IRAK 3, Ly96), and transcriptional regulation (zinc finger proteins) while M3.3 included genes encoding antigens to scavenger receptor proteins (CD36), coagulation (factor V), and lysosomal functions (LAMP2). A number of genes were significantly over-expressed in patients compared to controls in module M2.3 (“erythrocytes”) related to hemoglobin (hemoglobin alpha and gamma, erythrocyte membrane protein, erythroid factors) and module M3.5 (“undetermined”) including hemoglobin alpha and gamma proteins.

Conversely, there was significant under-expression of genes related to the adaptive immune response including those in M1.3 (“B cells”) such as genes encoding cell surface molecules CD19, CD22, CD72, CD79 and genes involved in immunoglobulin production, and in M2.8 (“T cells”) including CD6, CD96, ITK, and M2.1 (“cytotoxic cells”) (KLR subfamilies, Granulysin, Granzyme B) and genes encoding TNF family members. Module M1.8 (“undetermined”) included under-expression of factors involved in DNA replication and transcription (zinc finger protein genes) and cytokines (IL16); significant under-expressed genes in M3.8 (“undetermined”) also included multiple zinc finger proteins and TNF receptor-associated factors (TRAF5).

The PBMC gene list from *S. aureus*-infected patients was then analyzed in the test set of patients and module analysis validated the initial findings. Both the actual gene probes and the percentage of over or under-expressed genes per module in patients with *S. aureus* infection in the test set matched what had been demonstrated in the training set of patients, confirming the value and consistency of the PBMC transcriptional signature in patients with *S. aureus* infection **(** **Figure 2a** **)**.

### Confirming the robustness of the gene expression signature in S. aureus-infected patients

Twenty-five additional subjects were enrolled to further validate the PBMC gene expression profile of *S. aureus*-infected patients by confirming its reproducibility across microarray platforms. This validation set was comprised of 11 patients with invasive *S. aureus* infections and 14 age and sex-matched healthy controls. Transcript sequences from RefSeq were used to match corresponding valid gene probe sets on each platform. This allowed mapping of the 3,067 significant genes comprising the gene expression profile of PBMC of *S. aureus*-infected patients based on the Affymetrix gene probes to their corresponding 1,521 gene probes on the Illumina platform. This 1,521 gene list was then applied to the independent validation set of 9 patients and 9 controls using an unsupervised scheme that allowed clustering of samples based solely on intrinsic gene expression levels **(** **Figure 2b** **)**. Despite technical differences between the two platforms, all 9 patients with *S. aureus* infection clustered together (red horizontal bar) based on similarities in PBMC gene expression patterns alone.

Module level analyses of the 1,521 gene probes on the Illumina platform demonstrate similarities between the Affymetrix and the Illumina data. As illustrated in **Figure 2b**, 16 of 19 modules yielded concordant results in both the Illumina and Affymetrix platforms. Only modules M1.4, M3.5, and M3.6 did not show significant differences in Illumina and may be a result of representation of less gene probes in those modules. Correlation analyses of the average normalized values among the differentially expressed genes for each given module between both platforms was statistically significant (Spearman R = 0.76, p = 0.002). Thus, both unsupervised hierarchical clustering and modular analyses confirmed the robustness of the gene expression profile in PBMC of *S. aureus*-infected patients across both the Affymetrix and Illumina BeadChip microarray platforms in distinct groups of subjects.

### Decreased number of central memory CD4+ and CD8+ T cells in patients with S. aureus infections

To understand how the changes in blood transcriptional profiles in patients with *S. aureus* infection relate to changes in the numbers of the different immune cell populations, a detailed flow cytometry analysis was simultaneously conducted in PBMCs of a subset of patients with *S. aureus* (n = 11) and appropriate age-matched healthy controls (n = 13).

**Figure 3:**
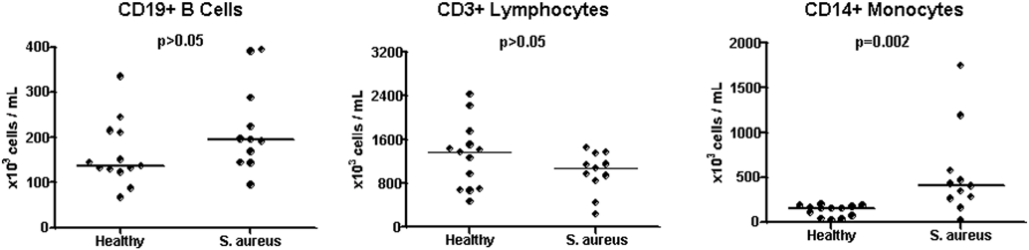
Peripheral blood monocytes are significantly expanded in patients with invasive *S. aureus* infections. PBMCs obtained from age-matched healthy donors (n = 13) and patients with *S. aureus* infection (n = 11) were analyzed by flow cytometry for the expression of CD19 (left panel), CD3 (middle), and CD14 (right) markers. Results are expressed as absolute number of cells per mL of blood. Bars represent median values. Mann-Whitney test was applied for statistical analysis.

**Figure 4:**
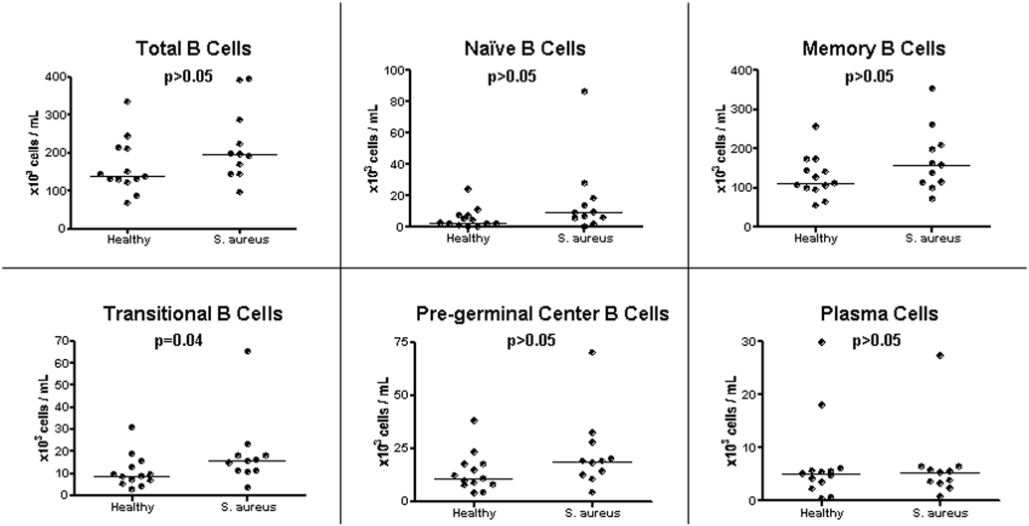
Analysis of the B cell compartment. PBMCs obtained from age-matched healthy donors (n = 13) and patients with *S. aureus* infection (n = 11) were stained by multicolor panel staining according to the expression of specific B cell markers. Results are expressed as absolute number of cells per mL of blood. Bars represent median values. Mann-Whitney test was applied for statistical analysis.

As illustrated in **Figure 3**, there were no significant differences in the total number of B cells and T cells between patients with *S. aureus* infection and healthy controls (p>0.05). Given the significant under-expression of genes related to these cell populations observed in the modular analysis (M1.3, M2.1, M2.8), further detailed flow analysis was performed in each lymphocyte compartment. Characterization of B cell subpopulations revealed no significant differences in the absolute number of naïve (CD19+/CD20+, IgD+, CD27−), memory (CD19+/CD20+/IgD+&−/CD27+), and plasma cells (CD19+/CD20−/CD27+&++/CD38++) between patients with *S. aureus* infections and healthy controls (**Figure 4**). Transitional B cells (CD19+/CD20+/CD24++/CD38++) were increased in patients with *S. aureus* infection compared with healthy controls (p = 0.04); there was also a trend (p = 0.06) toward increased numbers of pre-germinal B cells (CD19+/CD20+/CD27+/CD38++) in patients versus control subjects.

**Figure 5:**
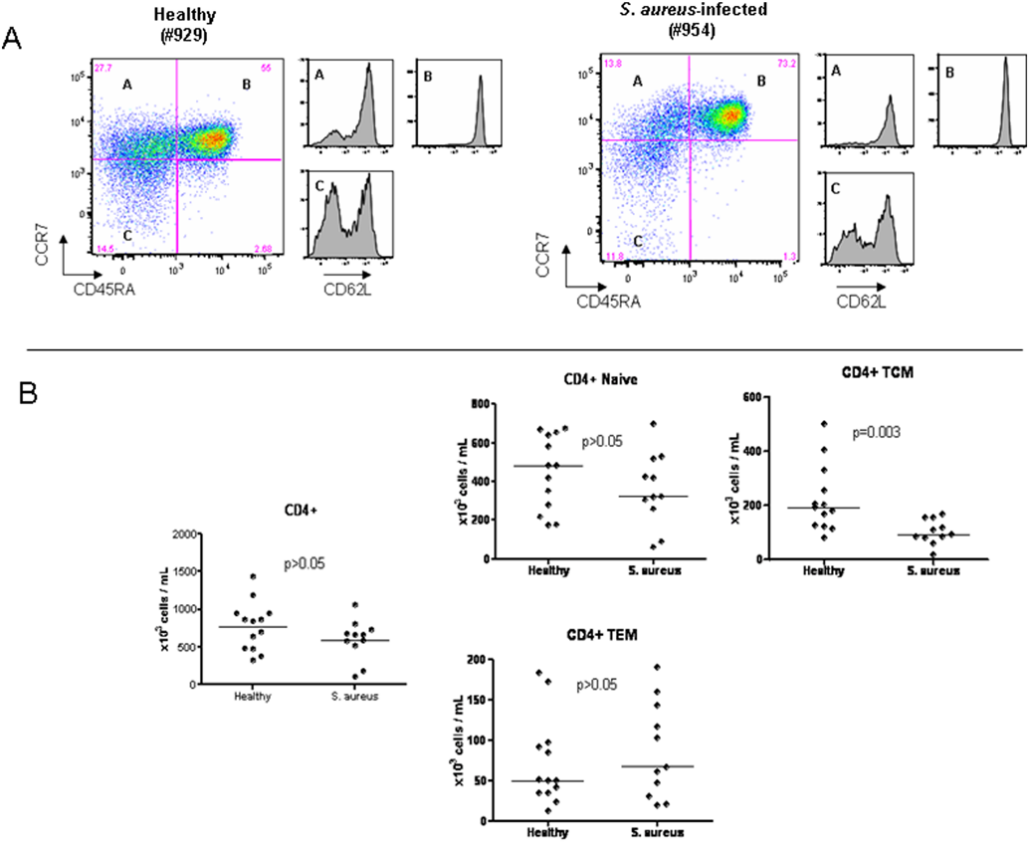
Analysis of the CD4+ T cell compartment. PBMCs obtained from age-matched healthy controls (n = 13) and patients with *S. aureus* infections (n = 11) were stained with CD3, CD4, CCR7, CD45RA, and CD62L antibodies and analyzed by flow cytometry. CD4 T cell subsets are labeled as (A) central memory T cells (TCM), (B) naïve T cells, and (C) effector memory T cells (TEM). (a) Flow cytometry plots of CD4 T cell subsets in a representative *S. aureus* patient and healthy control. CD62L expression is shown for each CD4 T cell subset. (b) Graphs show the absolute numbers of CD4 T cells and CD4 T cell subsets expressed as absolute number of cells per mL of blood. Horizontal lines represent median values. Statistical analysis was performed using a Mann-Whitney test.

**Figure 6:**
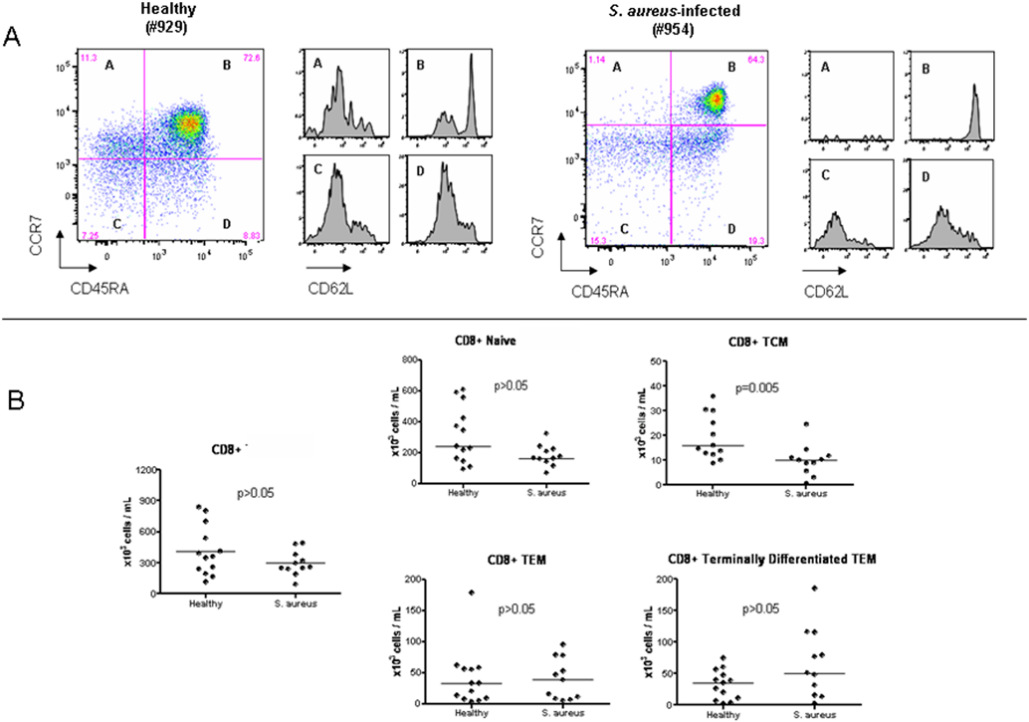
Analysis of the CD8+ T cell compartment. PBMCs obtained from patients with *S. aureus* infections (n = 11) and age-matched healthy controls (n = 13) were stained with CD3, CD8, CCR7, CD45RA, and CD62L antibodies and analyzed by flow cytometry. CD 8 T cells and CD 8 T cell subsets are labeled as: (A) central memory T cells (TCM); (B) naïve T cells, (C) effector memory T cells (TEM), and (D) terminally differentiated effector T cells (TEM). (a) Flow cytometry plots show CD8 T cell subsets in a representative *S. aureus* patient and healthy control. CD62L expression is shown for each CD8 T cell subset. (b) Graphs show the absolute numbers of CD8 T cells and CD8 T cell subsets. Results are expressed as absolute number of cells per mL of blood. Horizontal lines represent median values. Statistical analysis was performed using a Mann-Whitney test.

We then analyzed both CD4+ and CD8+ T cell and subpopulations of naïve and memory cells based on the expression of CD45RA, CD62L, and CCR7 as previously described [26]. As illustrated in **Figure 5** there were no differences in the absolute numbers of naïve CD4+ T cells and effector memory CD4+ T cells between the *S. aureus* patients and healthy controls. However, the absolute number of central memory CD4+ T cell was significantly reduced in *S. aureus* patients compared with controls (p = 0.003). With respect to CD8+ T cells, there were no differences between *S. aureus* patients and controls in the number of naïve, effector memory, and terminally differentiated CD8+ T cells (**Figure 6**). However, as was observed in the central memory CD4+ T cells, central memory CD8+ T cells were also significantly reduced in *S. aureus* patients compared with controls (p = 0.005). Thus, PBMCs of patients with invasive *S. aureus* infections demonstrate a significant reduction in the numbers of circulating central memory T cells.

### Monocyte expansion in patients with acute S. aureus infection

**Figure 7:**
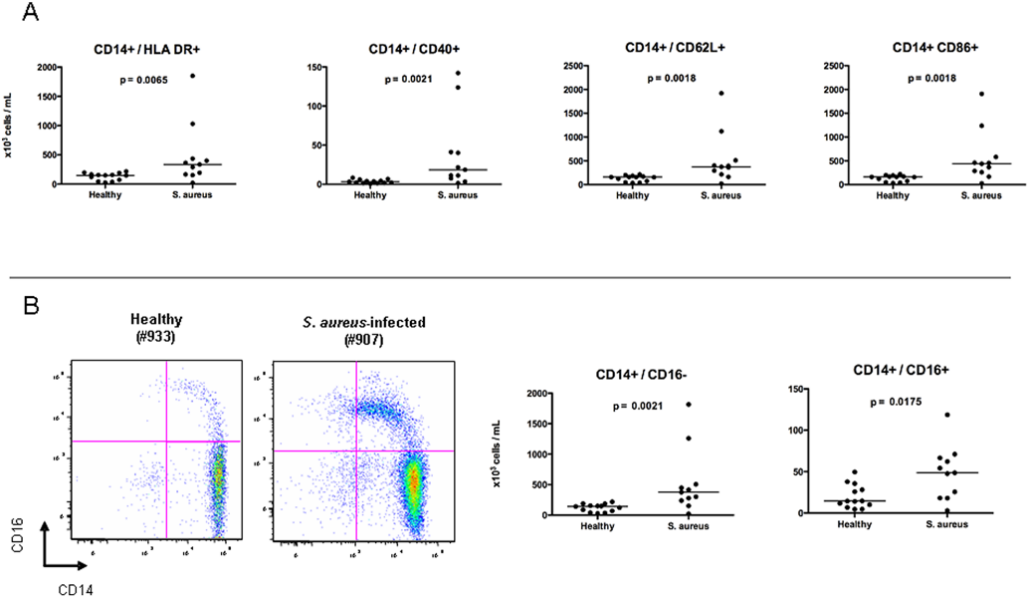
Analysis of monocyte compartment. PBMCs obtained from patients with *S. aureus* infections (n = 11) and age-matched healthy controls (n = 13) were stained with Monocytes expressing HLA DR, CD40, CD62L, CD16, and CD86 antibodies and analyzed by flow cytometry. (a) Graphs show the absolute monocyte cell numbers. Results are expressed as absolute number of cells per mL of blood. Horizontal lines represent median values. Statistical analysis was performed using a Mann-Whitney test. (b) There was a significant expansion of both the CD14+ CD16− and the CD14+ CD16+ monocyte populations as shown in a flow cytometry plot of a representative *S. aureus* patient (907) and healthy control (933).

As shown in **Figure 3**, there was a significant increase in the median total number of monocytes in PBMCs of patients with *S. aureus* infection when compared with healthy controls (p = 0.002). Detailed characterization of CD14+ subpopulations showed a significant increase of absolute number of monocytes expressing activation markers such as CD86, CD40, HLA-DR, and CD62L homing lymphocyte molecule in patients with *S. aureus* infection compared with controls (**Figure 7a**). Reanalysis of the CD14+ subpopulations was performed after removing patients with extreme values (patients 908, 952, or 960) in each subpopulation; *S. aureus* patients still demonstrated statistically significant increased numbers of CD14+ HLADR+ (p = 0.023), CD14+ CD40+ (p = 0.0076), CD14+ 62L+ (p = 0.0062), and CD14+86+ (p = 0.0062) when compared to healthy controls.

Based on the CD16 expression, circulating monocytes can be divided into functionally distinct subpopulations: CD14+CD16− monocytes and CD14+16+ monocytes. Patients with *S. aureus* infection showed a significant expansion of both CD14+16− (p = 0.0021) and CD14+16+ (p = 0.0175) monocytes compared with healthy controls (**Figure 7b**). These differences remained significant even when outliers were removed (CD14+16−, p = 0.0076; CD14+16−, p = 0.0324). Thus, PBMCs of patients with invasive *S. aureus* infections demonstrate a significant increase in the numbers of circulating monocytes.

### Gene expression levels correlate with specific immune cell populations

**Figure 8:**
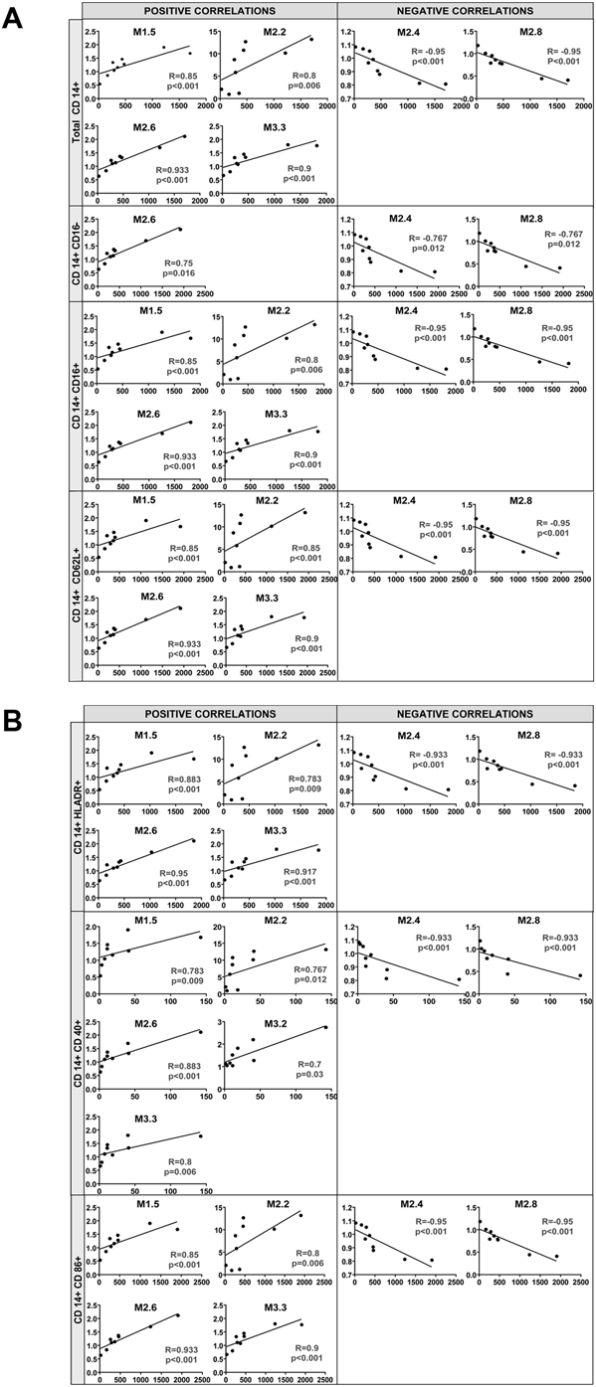
Significant correlations between absolute and subpopulations of monocytes and modular gene expression in patients with *S. aureus* infection. Correlation analyses were performed between the significant modules comprising the gene expression profile of PBMCs from *S. aureus*-infected patients and monocyte populations. Graphs represent the correlation (Spearman) between the average normalized average gene expression [log] significantly changed (Mann Whitney p<0.05) in the PBMCs of patients with *S. aureus* infection relative to the median gene expression of PBMCs in healthy controls in each significant module (y axis) and the corresponding absolute monocyte number and CD14+ subpopulations per mL of blood in *S. aureus*-infected patients (x axis).

To further characterize the relationship between the gene expression profiles in PBMCs of patients with *S. aureus* infections and the changes of the different immune cell populations, analyses were conducted on a group of patients (n = 9) and healthy controls (n = 9) in whom simultaneous gene expression and flow cytometry analyses were performed. To this end, the per module PBMC average gene expression obtained from patients with *S.aureus* infection relative to the healthy controls, were run on the Illumina platform and correlated with the absolute number of immune cells in each subject as measured by flow cytometry (**Table 5**). The most significant statistical correlations observed in patients with *S. aureus* infections were between the total number of CD14+ monocytes and CD14+ monocyte subpopulations and several gene expression modules (**Figure 8**). Absolute number of CD14+ monocytes significantly positively correlated with myeloid cell (M1.5 and M2.6), neutrophil (M2.2), and inflammation II (M3.3) modules (Spearman R≥0.8, p≤0.006). Total CD14+ monocyte cell numbers inversely correlated with the modules encoding genes for ribosomal proteins (M2.4) and T cells (M2.8). The number of CD14+16− cells positively correlated with M2.6 (“Myeloid”); CD14+16+ cells correlated with M2.6 (“Myeloid”). M1.5 (“Myeloid”), M2.2 (“Neutrophils”), and M3.3 (“Inflammation II”). The absolute number of CD14+CD16− and CD14+CD16+ cells inversely correlated with the ribosomal protein (M2.4) and T cell (M2.8) modules. Further correlations performed in the monocyte compartment of CD14+CD62L+, CD14+ HLA-DR+, CD14+CD40+, and CD14+CD86+ revealed significant positive correlations between these subpopulations and the myeloid module M2.6 and inverse correlations with ribosomal protein (M2.6) and T cell (M2.8) modules. Gene expression levels in modules related to neutrophils (M2.2) and inflammation module II (M3.3) positively correlated with CD14+CD62L+, CD14+ HLA-DR+, CD14+CD40+, and CD14+CD86+ cell numbers.

**Table 5:** Correlation of gene expression levels and absolute number of immune cells in patients with *S. aureus* infections.

FACS Cell Marker	Module	Assigned Immune Function	Correlation (Spearman)	p-value
**CD19**	*none*			
**CD3**	*none*			
**CD4**	M1.5	Myeloid	−0.683	0.0361
	M2.6	Myeloid	−0.650	0.05
	M3.3	Inflammation II	−0.683	0.0361
**CD8**	M3.1	Interferon	0.65	
**NK**	*none*			
**CD14**	M1.3	B cells	−0.633	0.0583
	M1.5	Myeloid	0.85	<0.001
	M2.2	Neutrophils	0.8	0.006
	M2.4	Ribosomal	−0.95	<0.001
	M2.6	Myeloid	0.933	<0.001
	M2.8	T cells	−0.95	<0.001
	M3.3	Inflammation II	0.9	<0.001

FACS = fluorescence-activated cell sorting; NK = Natural Killer cell.

With respect to the T cell compartment, the number of total CD4+ T cells inversely correlated with the myeloid (M1.5 and M2.6) and inflammation (M3.3) modules (p = 0.0361) (**Table 5**). Numbers of naïve CD4+ T cells positively correlated with the modules related to ribosomal proteins (M2.4) and T cells (M3.8), while inversely correlating with the M2.6 (Myeloid) and inflammation (M3.2 and M3.3) modules. The number of CD4+ T central memory cells (TCM) correlated inversely with myeloid (M1.5 and M2.6), neutrophil (M2.2), and inflammation (M3.3) modules, but positively with the ribosomal protein (M2.4), T cell (M2.8), and interferon (M3.1) modules. CD8+ T central memory cell numbers correlated inversely with myeloid modules (M1.5 and M2.6) and inflammation (M3.3) modules; conversely, CD8+ T effector memory cells correlated positively with cytotoxic cell module (M2.1) and undetermined (M1.8 and M3.7) modules.

There were no significant correlations between modular gene expression levels and CD4+ T effector memory, CD8+ naïve, and CD8+ terminally differentiated T cells. There were no significant correlations between the total number of CD19+ B cells, nor B cell subpopulations such as naïve B cells, memory B cells, transitional B cells, pre-germinal center B cells, and plasma cells B cell and modular gene expression patterns (**Table S2**).

## Discussion

The versatility of *S. aureus* has allowed emergence of highly resistant and virulent bacterial strains in the community [27]. Its pathogenicity is in part due to its repertoire of virulence factors and proclivity for tissue and endovascular invasion, destruction, and dissemination while simultaneously evading multiple components of the innate immune system and secreting immunomodulatory proteins that compromise both humoral and cell-mediated immunity [7], [27], [28]. Very little is known about the relationship between the human host and *S. aureus* during invasive infections, leading us to carry out the present study. We chose to apply a systems biology approach utilizing both gene expression microarray profiling and corresponding flow cytometry analyses to allow for a comprehensive assessment of the immunopathogenesis of the disease and interaction with the host.

By conducting several different step-wise analyses, the gene expression profile of patients with invasive *S. aureus* infections was defined and its robustness validated among distinct patient populations and across microarray platforms. Modular analysis demonstrated significant activation of host genes related to the innate immune response with increased expression of genes related to inflammatory processes and cells of the myeloid lineage and significant under-expression of genes related to the adaptive immune response. Although the over-expression of the innate immune response genes was not unexpected [28], [29], the striking and consistent decreased expression of B and T cell-related genes was less anticipated.

There is limited and conflicting information regarding the numbers of lymphocyte populations in patients with acute *S. aureus* infections [30]–[33]. In one study, patients with *S. aureus* and *S. pneumoniae* sepsis showed significantly decreased numbers of CD4+ and CD8+ T cells, and NK cells [34] while in another, patients with MRSA superantigen-associated glomerulonephritis showed increased numbers of DR+ CD4+ and CD8+ T cells and NK cells [35]. Flow cytometry analyses were performed in our study subjects to better understand whether the changes observed in the gene expression patterns simply reflected alterations in immune cell numbers. Despite significant under-expression of T and B cell-related genes observed in our patients with *S. aureus* infections, there were no differences in the absolute numbers of total B and T cells between infected patients and healthy controls. No consistent significant differences were seen between patients and controls across B cell subpopulations evaluated, however there were possible trends in transitional and pre-germinal B cells that will require analysis in a larger sample size.

Detailed analysis of the T cell sub-populations revealed decreased numbers of both central memory CD4+ T cells and CD8+ T cells, but no differences in the other T cell subsets in patients with *S. aureus* infection. Central memory T cells have been shown to have a high proliferative potential and to demonstrate *in vivo* persistence [36], [37]. Our results demonstrate a decreased number of central memory T cells, suggesting a possible reorganization of the circulating T cell compartment that may also explain the reduced expression of T cell-related genes. With increased expression of CCR7 and CD62L, central memory T cells are programmed to preferentially migrate to lymphoid tissues to interact with other T and B cells in establishing a repertoire of effector functions against the invading pathogen [26]. One possible explanation then, for the significant reduction in the numbers of circulating central memory T cells seen in our subjects with *S. aureus* infection may be central memory T cell homing to these secondary lymph organs. *S. aureus* expresses factors that promote its immune evasion and could also elicit this cellular imbalance. *In vitro* observations have demonstrated a shift from central memory to effector memory T cells in the presence of *S. aureus* enterotoxin [26]. Secretion of other superantigens by *S. aureus*, such as Map/Eap (MHC Class II analogous protein/extracellular adherence protein), bind to T-cell receptors and directly thwart T cell responses by reducing T cell proliferation, altering effector functions, and stimulating apoptosis [7].

Alternatively, the under-expression of T cell genes may be due to an increase in other cell populations. Indeed, the monocyte compartment, total number and subpopulations, was significantly expanded in patients with *S. aureus* infection and correlated with gene expression levels, providing the cellular component to the increased expression of innate immune genes. Based on CD16 expression, circulating monocytes can be divided into functionally distinct subpopulations CD14+CD16− and CD14+16+ monocytes, which may have distinct roles in the innate immune response [38]. In the *S. aureus* patients, there was a significant expansion of both of these monocyte subsets. Furthermore, the high levels of expression of genes generally associated with neutrophil functions, such as defensins, lactotransferrins, CAMP, and elastase, as seen in module M2.2, may originate from the CD14+16+ (CD62L−) monocytes subset, as has been recently described [38]. Although significant numbers of neutrophils are not generally present in PBMCs, low-density neutrophils have been demonstrated and accounted for over-expression of neutrophil genes in patients with SLE [39]. Despite using similar technical approaches, we were not able to detect neither these low-density immature neutrophils nor high-density neutrophils that could result from contamination in our samples. Studies with purified cell subsets will be necessary to understand the distinct contribution from each cell subtype.

These results demonstrate the value of gene expression profiling in combination with flow cytometry as a new strategy to study disease pathogenesis within the clinical context. This study provides information on the profound dysregulation of both the innate and adaptive immune responses induced by invasive *S. aureus* infections and opens new avenues for improving our understanding of this infection that may lead to improved biomarker discovery and therapeutic interventions.

## Materials and Methods

### Ethics Statement

This study was conducted according to the principles expressed in the Declaration of Helsinki. The study was approved by the Institutional Review Boards of the University of Texas Southwestern Medical Center and Children's Medical Center of Dallas (IRB #0802-447) and Baylor Institute of Immunology Research (BIIR, IRB # 002-141). Informed consent was obtained from legal guardians and informed assent was obtained from patients 10 years of age and older prior to any study-related procedure.

### Patient information

Blood samples were collected from 77 children: 53 patients with *S. aureus* infection and 24 healthy controls. Children with suspected or proven polymicrobial infections, underlying chronic disease, immunodeficiency, or those who received steroids or other immunomodulatory therapies were excluded. Control samples were obtained from healthy children undergoing elective surgical procedures and at healthy outpatient clinic visits. Nasopharyngeal viral cultures were obtained in both patients and controls to exclude viral co-infections. Children hospitalized with acute *S. aureus* infections were offered participation in the study after microbiologic confirmation of the diagnosis by standard bacterial culture of blood or tissue specimens. Patients were analyzed in 3 groups: training (20 *S. aureus*, 10 healthy), test (22 *S. aureus*, 10 healthy), and validation sets (11 *S. aureus*, 14 healthy).

### Sample collection

Blood samples (3–8 mL) were collected in acid-citrate-dextrose tubes (ACD tubes, BD Vacutainer, Franklin Lakes, NJ) and delivered to the laboratory at room temperature for processing. Peripheral blood mononuclear cells (PBMCs) were isolated by density gradient centrifugation using Ficoll-hypaque technique and lysed in RLT reagent (Qiagen, Valencia, CA) with β-mercaptoethanol (BME) and stored at -80°C until processing. Samples were run in batches by the same laboratory team to ensure standardization of quality and handling of samples. Total RNA was isolated using the RNeasy Mini Kit (Qiagen, Valencia, CA) per the manufacturer's instructions and RNA integrity was assessed using an Agilent 2100 Bioanalyzer (Agilent, Palo Alto, CA).

### Microarray procedures

#### Affymetrix

From 2–5 micrograms of total RNA, double-stranded cDNA was generated as a template for single-round *in vitro* transcription with biotin-labeled nucleotides using the Affymetrix cDNA Synthesis and In Vitro Transcription kits (Affymetrix Inc., Santa Clara, CA). Biotinylated cRNA targets were then purified (Sample Cleanup Module, Affymetrix) and hybridized to the Affymetrix HG-U133A and B GeneChip arrays (Affymetrix Inc., Santa Clara, CA) according to the manufacturer's standard protocols.

#### Affymetrix Gene Chips

Arrays were scanned using a laser confocal scanner (Agilent). Global gene expression analysis was carried out using the Affymetrix HG-U133A and U133B GeneChips. The HG-U133 set contains 44,760 probe sets representing >39,000 transcripts derived from ∼33,000 human genes. Raw signal intensity values were normalized to the mean intensity of all measurements per gene chip and scaled to a target intensity value of 500 using the MAS 5.0 global scaling method to adjust for possible chip-to-chip variations in hybridization intensities (GeneChip Operating System version 1.0). Data was imported into GeneSpring software (version 7.3.1, Agilent) to perform the gene expression analyses, statistical testing, hierarchical clustering, and classification of samples.

*Illumina*: Double-stranded cDNA was obtained from 200 ng of total RNA and after *in vitro* transcription underwent amplification and labeling steps according to the manufacturer's instructions. 1.5 µg of amplified biotin-labeled cRNA was hybridized to the Illumina Sentrix Hu6 BeadChips according to the sample labeling procedure recommended by Illumina. (Ambion, Inc, Austin, TX).

*Illumina BeadChips*: The Sentrix Hu6 BeadChips consist of 50mer oligonucleotide probes attached to 3-µm beads within microwells on the surface of the glass slide representing 48,687 probes. Slides were scanned on Illumina BeadStation 500 and Beadstudio software was used to assess fluorescent hybridization signals.

### Microarray Data Analysis

Using the Genespring™ software program (Agilent), the expression value for each gene per individual subject's sample was normalized to the median expression value of the same gene in samples from healthy controls. Class comparison analyses were performed on probe sets present in at least 75% of samples in each group (quality control (QC) probes). Non-parametric statistical testing (Wilcoxon-Mann-Whitney U-test; p<0.01 for class comparisons; p<0.05 for modular analyses with no multiple test corrections) was used to rank genes based on their ability to discriminate among pre-specified groups of patients. Final lists of significantly changed genes used in class comparisons were filtered to include only those transcripts that showed a 1.25-fold or greater fold change in expression level relative to the control group. Hierarchical clustering was applied to order genes according to expression levels. The list of most highly ranked genes from the *S. aureus* biosignature was created by ranking genes with the highest fold change difference and the most significantly different genes (by p value) between healthy controls and patients with infection.

For the cross microarray platform validation part of the analysis we performed a two-step procedure. First, we examined the genes that defined the *S. aureus* biosignature obtained on the test and training set of subjects analyzed on the Affymetrix platform in a new set of 18 subjects (validation set) and run on the lllumina microarray platform. Transcript sequences from RefSeq were used to perfectly match corresponding valid gene probe sets on each platform; Affymetrix probe set data encoded by Mage-ML files in XML format were matched by GenBank accession numbers and reference sequence transcripts to the Illumina manifest probe mapping file (http://www.switchtoi.com/probemapping.ilmn) allowing for mapping of the significant Affymetrix gene probes to their corresponding gene probes on the Illumina platform. In the second step of the analysis, the Illumina gene list was applied to an independent validation set of subjects using an unsupervised scheme that allowed clustering of samples based solely on intrinsic gene expression levels.

### Transcriptional Module Analysis

A detailed account of this module-based data mining analysis strategy has been reported elsewhere [25]. Briefly, this is a systems-scale strategy for microarray analysis that has identified transcriptional modules formed by genes coordinately expressed across multiple disease data sets thus allowing functional interpretation of the microarray data into biologically useful information. Analysis of linear correlations between gene expression levels per module in the different microarray platforms (Affymetrix vs. Illumina) and between the average gene expression levels per module (from the validation set run on Illumina) and the individual immune cell numbers per patient (calculated by Flow cytometry) were performed using a non-parametric statistical correlation analysis test (Spearman).

### Flow cytometry

PBMCs were isolated by density gradient centrifugation using Ficoll-hypaque technique from blood samples (3–8 mL) collected in ACD tubes. One million PBMCs were washed and incubated with conjugated antibodies for 15 minutes at room temperature, in dark conditions according to multi-color staining panels described below. Cells were washed with phosphate buffer and then fixed with 2% paraformaldehyde. Samples were run on a BD LSRII flow cytometer (BD BioScience, San Jose, CA); 50,000 events were acquired with the BD FACSDiva™ software according to the lymphocyte gate and analyzed using FlowJo™ software (Tree Star, Inc).

Multicolor staining panels included: B cell panel: CD19 (ECD, Beckman-Coulter), CD20 (Pe-Cy5, BD Pharmingen), CD24 (PE, BD Pharmingen), CD27 (APC, BD Pharmingen), CD38 (Pe-Cy7, BD Biosciences) and IgD (FITC, SouthernBiotech). T cell panel: CD3 (Alexa700, BD Pharmingen), CD4 (Pacific Blue, Invitrogen), CD8 (APC-Cy7, BD Pharmingen), CD45RA (ECD, Beckman-Coulter), CD62L (Pe-Cy5, BD Pharmingen), CCR7 (Pe-Cy7, BD Pharmingen). Monocyte panel: CD14 (Pacific Blue, BD Pharmingen), CD16 (APC, Invitrogen), CD40 (PE, BD Pharmingen), CD86 (FITC, BD Pharmingen), HLA-DR (APC-Cy7, BD Pharmingen) and CD62L (ECD, Beckman-Coulter). Isotype controls: IgG1 (FITC, BD Biosciences), IgG1 (PE, BD Biosciences), IgG1 (ECD, Beckman-Coulter), IgG1 (Pe-Cy5, BD Biosciences), IgG1 (Pe-Cy7, BD Biosciences), IgG1 (APC, BD Biosciences), IgG1 (Alexa 700, BD Biosciences), IgG1 (APC-Cy7, BD Biosciences), IgG1 (Pacific Blue, Invitrogen).

Cell populations were analyzed based on the following markers: B cells: CD19; Naïve B cells: CD19+/CD20+, IgD+, CD27−; Memory B cells: CD19+/CD20+/IgD+&−/CD27+; Plasma cells: CD19+/CD20−/CD27+&++/CD38++; Transitional B cells: CD19+/CD20+/CD24++/CD38++; Pre-germinal center B cells: CD19+/CD20+/CD27+/CD38++; T cells: Naïve T cells: CD3+/CD4+ or CD8+/CD45RA+/CCR7+; Central memory T cells: CD3+/CD4+ or CD8+/CD45RA−/CCR7+; Effector memory T cells: CD3+/CD4+ or CD8+/CD45RA−/CCR7−; CD8+ terminally differentiated T cells: CD3+/CD8+/CD45RA+/CCR7−.

### Online Supporting Information

**Table S1** provides the functional interpretation of the transcriptional modules including module, number of probe tests evaluated, key words included in data mining, and immune interpretation. **Table S2** details the results of the non-parametric correlation analyses performed between B and T cell populations and module analyses. **Figure S1** provides graphic representation and detailed information regarding significantly differently expressed genes in patients with S. aureus infection versus healthy control per module.

### Accession Numbers

The microarray data used in this study is deposited in NCBI Gene Expression Omnibus (GEO).

## Supporting Information

Table S1 Functional interpretation of transcriptional modules.
(0.06 MB DOC)

Table S2 Correlations of B and T cell subsets with transcriptional modules.
(0.05 MB DOC)

Figure S1 Module analysis of the gene expression profile in patients with *S. aureus* infections. Graphs represent the average gene expression level (y axis) significantly changed (Mann Whitney p<0.05) in patients with *S. aureus* infections (x axis, red horizontal bar) versus healthy controls (x axis, green horizontal bar) in each of the 19 significant modules (M) comprising the gene expression profile seen in PBMCs of *S. aureus*-infected patients. A detailed list citing the gene probe, pvalue, and description of the significant transcript is noted.
(0.57 MB DOC)
