# Supplementary material for: Enhanced Monocyte Response and Decreased Central Memory T Cells in Children with Invasive Staphylococcus aureus Infections
Source: PLoS One. 2009 May 8;4(5):e5446. doi: 10.1371/journal.pone.0005446 (PMC2676512; doi:10.1371/journal.pone.0005446)
Supplement: Table S1 — Functional interpretation of transcriptional modules. (0.06 MB DOC) [file pone.0005446.s001.doc]

**Table S1. Functional interpretation of transcriptional modules.**

| **Module I.D.** | **Number of probe sets** | **Keyword selection** | **Assessment** |
| --- | --- | --- | --- |
| **M 1.1** | 76 | Ig, Immunoglobulin, Bone, Marrow, PreB, IgM,Mu. | **Plasma cells.** Includes genes coding for Immunoglobulin chains (*e.g*. IGHM, IGJ, IGLL1, IGKC, IGHD) and the plasma cell marker CD38. |
| **M 1.2** | 130 | Platelet, Adhesion, Aggregation, Endothelial, Vascular | **Platelets.** Includes genes coding for platelet glycoproteins (ITGA2B, ITGB3, GP6, GP1A/B), and platelet-derived immune mediators such as PPPB (pro-platelet basic protein) and PF4 (platelet factor 4). |
| **M 1.3** | 80 | Immunoreceptor , BCR, B-cell, IgG | **B-cells.** Includes genes coding for B-cell surface markers (CD72, CD79A/B, CD19, CD22) and other B-cell associated molecules: Early B-cell factor (EBF), B-cell linker (BLNK) and B lymphoid tyrosine kinase (BLK). |
| **M 1.4** | 132 | Replication, Repression, Repair, CREB, Lymphoid, TNF-alpha | **Undetermined.** This set includes regulators and targets of cAMP signaling pathway (JUND, ATF4, CREM, PDE4, NR4A2, VIL2), as well as repressors of TNF-alpha mediated NF-KB activation (CYLD, ASK, TNFAIP3). |
| **M 1.5** | 142 | Monocytes, Dendritic, MHC, Costimulatory, TLR4, MYD88 | **Myeloid lineage.** Includes molecules expressed by cells of the myeloid lineage (CD86, CD163, FCGR2A), some of which being involved in pathogen recognition (CD14, TLR2, MYD88). This set also includes TNF family members (TNFR2, BAFF). |
| **M 1.6** | 141 | Zinc, Finger, P53, RAS | **Undetermined.** This set includes genes coding for signaling molecules, *e.g*. the zinc finger containing inhibitor of activated STAT (PIAS1 and PIAS2), or the nuclear factor of activated T-cells NFATC3. |
| **M 1.7** | 129 | Ribosome, Translational, 40S, 60S, HLA | **MHC/Ribosomal proteins.** Almost exclusively formed by genes coding MHC class I molecules (HLA-A,B,C,G,E)+ Beta 2-microglobulin (B2M) or Ribosomal proteins (RPLs, RPSs). |
| **M 1.8** | 154 | Metabolism, Biosynthesis, Replication, Helicase | **Undetermined.** Includes genes encoding metabolic enzymes (GLS, NSF1, NAT1) and factors involved in DNA replication (PURA, TERF2, EIF2S1). |
| **M 2.1** | 95 | NK, Killer, Cytolytic, CD8, Cell-mediated, T-cell, CTL, IFN-g | **Cytotoxic cells.** Includes cytotoxic T-cells amd NK-cells surface markers (CD8A, CD2, CD160, NKG7, KLRs), cytolytic molecules (granzyme, perforin, granulysin), chemokines (CCL5, XCL1) and CTL/NK-cell associated molecules (CTSW). |
| **M 2.2** | 49 | Granulocytes, Neutrophils, Defense, Myeloid, Marrow | **Neutrophils.** This set includes innate molecules that are found in neutrophil granules (Lactotransferrin: LTF, defensin: DEAF1, Bacterial Permeability Increasing protein: BPI, Cathelicidin antimicrobial protein: CAMP). |
| **M 2.3** | 148 | Erythrocytes, Red, Anemia, Globin, Hemoglobin | **Erythrocytes.** Includes hemoglobin genes (HGBs) and other erythrocyte-associated genes (erythrocytic alkirin:ANK1, Glycophorin C: GYPC, hydroxymethylbilane synthase: HMBS, erythroid associated factor: ERAF). |
| **M 2.4** | 133 | Ribonucleoprotei n, 60S, nucleolus, Assembly, Elongation | **Ribosomal proteins.** Including genes encoding ribosomal proteins (RPLs, RPSs), Eukaryotic Translation Elongation factor family members (EEFs) and Nucleolar proteins (NPM1, NOAL2, NAP1L1). |
| **M 2.5** | 315 | Adenoma, Interstitial, Mesenchyme, Dendrite, Motor | **Undetermined.** This module includes genes encoding immune-related (CD40, CD80, CXCL12, IFNA5, IL4R) as well as cytoskeleton-related molecules (Myosin, Dedicator of Cytokenesis, Syndecan 2, Plexin C1, Distrobrevin). |
| **M 2.6** | 165 | Granulocytes, Monocytes, Myeloid, ERK, Necrosis | **Myeloid lineage.** Includes genes expressed in myeloid lineage cells (IGTB2/CD18, Lymphotoxin beta receptor, Myeloid related proteins 8/14 Formyl peptide receptor 1), such as Monocytes and Neutrophils. |
| **M 2.7** | 71 | No keywords extracted. | **Undetermined.** This module is largely composed of transcripts with no known function. Only 20 genes associated with literature, including a member of the chemokine-like factor superfamily (CKLFSF8). |
| **M 2.8** | 141 | Lymphoma, T-cell, CD4, CD8, TCR, Thymus, Lymphoid, IL2 | **T-cells.** Includes T-cell surface markers (CD5, CD6, CD7, CD26, CD28, CD96) and molecules expressed by lymphoid lineage cells (lymphotoxin beta, IL2-inducible T-cell kinase, TCF7, T-cell differentiation protein mal, GATA3, STAT5B). |
| **M 2.9** | 159 | ERK, Transactivation, Cytoskeletal, MAPK, JNK | **Undetermined.** Includes genes encoding molecules that associate to the cytoskeleton (Actin related protein 2/3, MAPK1, MAP3K1, RAB5A). Also present are T-cell expressed genes (FAS, ITGA4/CD49D, ZNF1A1). |
| **M 2.10** | 106 | Myeloid, Macrophage, Dendritic, Inflammatory, Interleukin | **Undetermined.** Includes genes encoding for Immune-related cell surface molecules (CD36, CD86, LILRB), cytokines (IL15) and molecules involved in signaling pathways (FYB, TICAM2-Toll­like receptor pathway). |
| **M 2.11** | 176 | Replication, Repress, RAS, Autophosphoryla tion, Oncogenic | **Undetermined.** Includes kinases (UHMK1, CSNK1G1, CDK6, WNK1, TAOK1, CALM2, PRKCI, ITPKB, SRPK2, STK17B, DYRK2, PIK3R1, STK4, CLK4, PKN2) and RAS family members (G3BP, RAB14, RASA2, RAP2A, KRAS). |
| **M 3.1** | 122 | ISRE, Influenza, Antiviral, IFN-gamma, IFN-alpha, Interferon | **Interferon-inducible.** This set includes interferon-inducible genes: antiviral molecules (OAS1/2/3/L, GBP1, G1P2, EIF2AK2/PKR, MX1, PML), chemokines (CXCL10/IP-10), signaling molecules (STAT1, STAt2, IRF7, ISGF3G). |
| **M 3.2** | 322 | TGF-beta, TNF, Inflammatory, Apoptotic, Lipopolysacchari de | **Inflammation I.** Includes genes encoding molecules involved in inflammatory processes (*e.g*. IL8, ICAM1, C5R1, CD44, PLAUR, IL1A, CXCL16), and regulators of apoptosis (MCL1, FOXO3A, RARA, BCL3/6/2A1, GADD45B). |
| **M 3.3** | 276 | Inflammatory, Defense, Lysosomal, Oxidative, LPS | **Inflammation II.** Includes molecules inducing or inducible by inflammation (IL18, ALOX5, ANPEP, AOAH, HMOX1, SERPINB1), as well as lysosomal enzymes (PPT1, CTSB/S, NEU1, ASAH1, LAMP2, CAST). |
| **M 3.4** | 325 | Ligase, Kinase, KIP1, Ubiquitin, Chaperone | **Undetermined.** Includes protein phosphatases (PPP1R12A, PTPRC, PPP1CB, PPM1B) and phosphoinositide 3­kinase (PI3K) family members (PIK3CA, PIK32A, PIP5K3). |
| **M 3.5** | 22 | No keyword extracted | **Undetermined.** Composed of only a small number of transcripts. Includes hemoglobin genes (HBA1, HBA2, HBB). |
| **M 3.6** | 288 | Ribosomal, T-cell, Beta­catenin | **Undetermined.** This set includes mitochondrial ribosomal proteins (MRPLs, MRPs), mitochondrial elongations factors (GFM1/2), Sortin Nexins (SN1/6/14) as well as lysosomal ATPases (ATP6V1C/D). |
| **M 3.7** | 301 | Spliceosome, Methylation, Ubiquitin | **Undetermined.** Includes genes encoding proteasome subunits (PSMA2/5, PSMB5/8); ubiquitin protein ligases HIP2, STUB1, as well as components of ubiqutin ligase complexes (SUGT1). |
| **M 3.8** | 284 | CDC, TCR, CREB, Glycosylase | **Undetermined.** Includes genes encoding enzymes: aminomethyltransferase, arginyltransferase, asparagines synthetase, diacylglycerol kinase, inositol phosphatases, methyltransferases, helicases… |
| **M 3.9** | 260 | Chromatin, Checkpoint, Replication, Transactivation | **Undetermined.** Includes genes encoding kinases (IBTK, PRKRIR, PRKDC, PRKCI) and phosphatases (*e.g*. PTPLB, PPP2CB/3CB, PTPRC, MTM1, MTMR2). |
