# Supplementary material for: Enhanced Monocyte Response and Decreased Central Memory T Cells in Children with Invasive Staphylococcus aureus Infections
Source: PLoS One. 2009 May 8;4(5):e5446. doi: 10.1371/journal.pone.0005446 (PMC2676512; doi:10.1371/journal.pone.0005446)
Supplement: Table S2 — Correlations of B and T cell subsets with transcriptional modules. (0.05 MB DOC) [file pone.0005446.s002.doc]

**Table S2: Correlations of B and T cell subsets with transcriptional modules**

| **FACS Cell Marker** | **Module** | **Assigned Immune Function** | **Correlation (Spearman)** | **p-value** |
| --- | --- | --- | --- | --- |
| **CD4, naïve** | M2.4 | Ribosomal | 0.683 | 0.036 |
|  | M2.6 | Myeloid | -0.65 | 0.05 |
|  | M2.8 | T cells | 0.683 | 0.036 |
|  | M3.2 | Inflammation I | -0.65 | 0.05 |
|  | M3.3 | Inflammation II | -0.7 | 0.03 |
| **CD4 TCM** | M1.5 | Myeloid | -0.783 | 0.009 |
|  | M2.2 | Neutrophils | -0.667 | 0.043 |
|  | M2.4 | Ribosomal | 0.65 | 0.05 |
|  | M2.6 | Myeloid | -0.717 | 0.025 |
|  | M2.8 | T cells | 0.65 | 0.05 |
|  | M3.1 | Interferon | 0.7 | 0.03 |
|  | M3.3 | Inflammation II | -0.8 | 0.006 |
| **CD4 TEM** |  |  | *none* |  |
| **CD8, naïve** |  |  | *none* |  |
| **CD 8 TCM** | M1.5 | Myeloid | -0.767 | 0.012 |
|  | M2.6 | Myeloid | -0.683 | 0.036 |
|  | M3.3 | Inflammation II | -0.783 | 0.009 |
| **CD8 TEM** | M1.8 | Undetermined | 0.65 | 0.05 |
|  | M2.1 | Cytotoxic cells | 0.667 | 0.043 |
|  | M3.7 | Undertermined | 0.683 | 0.036 |
| **CD8-terminally**  **differentiated** |  |  | *none* |  |
| **B cells, naïve** |  |  | *none* |  |
| **Memory B** |  |  | *none* |  |
| **Transitional B** |  |  | *none* |  |
| **Pre-germinal B** |  |  | *none* |  |
| **Plasma B** |  |  | *none* |  |

FACS= fluorescence-activated cell sorting; TCM= central memory T cell; TEM = effector memory T cell
