# Supplementary material for: Enhanced Monocyte Response and Decreased Central Memory T Cells in Children with Invasive Staphylococcus aureus Infections
Source: PLoS One. 2009 May 8;4(5):e5446. doi: 10.1371/journal.pone.0005446 (PMC2676512; doi:10.1371/journal.pone.0005446)
Supplement: Figure S1 — Module analysis of the gene expression profile in patients with S. aureus infections. Graphs represent the average gene expression level (y axis) significantly changed (Mann Whitney p<0.05) in patients with S. aureus infections (x axis, red horizontal bar) versus healthy controls (x axis, green horizontal bar) in each of the 19 significant modules (M) comprising the gene expression profile seen in PBMCs of S. aureus-infected patients. A detailed list citing the gene probe, pvalue, and description of the significant transcript is noted. (0.57 MB DOC) [file pone.0005446.s003.doc]

**Gene Probe** **p-Value** **Description**

230942_at 0.0006 Chemokine-like factor super family 5

208792_s_at0.000827 Clusterin

216956_s_at0.000827 Integrin, alpha 2b

31874_at 0.000827 Growth arrest-specific 2 like 1

206493_at 0.00132 Integrin, alpha 2b

201108_s_at0.00154 Thrombospondin 1

208791_at 0.00154 Clusterin

206698_at 0.00207 Kell blood group precursor

207808_s_at0.00207 Protein S (alpha)

206167_s_at0.00278 Rho GTPase activating protein 6

203305_at 0.0032 Coagulation factor XIII, A1 polypeptide

204628_s_at0.0032 Integrin, beta 3

209806_at 0.00638 Histone 1, H2bk

210387_at 0.00638 Homo sapiens cDNA clone IMAGE:2989839, with

204081_at 0.00944 Neurogranin

204627_s_at0.00944 Integrin, beta 3

206655_s_at0.0122 Glycoprotein Ib (platelet)

217963_s_at0.0122 Nerve growth factor receptor (TNFRSF16)

203680_at 0.0138 Protein kinase, cAMP-dependent, regulatory, type II, beta

205127_at 0.0138 Prostaglandin-endoperoxide synthase 1

37965_at 0.0155 Parvin, beta

203585_at 0.0175 Zinc finger protein 185

206049_at 0.0175 Selectin P

230645_at 0.0175 FERM domain containing 3

242094_at 0.0175 Full length insert cDNA clone YR40C10

205128_x_at0.0197 Prostaglandin-endoperoxide synthase 1

226018_at 0.0197 zd56c09.s1 Soares_fetal_heart_NbHH19W Homo sapiens

34408_at 0.0197 Reticulon 2

212077_at 0.0222 Caldesmon 1

203817_at 0.0249 Guanylate cyclase 1, soluble, beta 3

225354_s_at0.0249 match: proteins O75368 P55822 Q9BPY5 Q9BRB8 Q9WUZ7

48031_r_at0.0249 Chromosome 5 open reading frame 4

214146_s_at0.0278 Pro-platelet basic protein

204069_at 0.0311 Meis1, myeloid ecotropic viral integration site 1 homolog

202729_s_at0.0347 Latent transforming growth factor beta binding protein 1

214039_s_at0.0347 Lysosomal associated protein transmembrane 4 beta

207206_s_at0.0387 Arachidonate 12-lipoxygenase

224823_at 0.0387 Myosin, light polypeptide kinase

207389_at 0.043 Glycoprotein Ib (platelet), alpha polypeptide

202708_s_at0.0477 Histone 2, H2be

214974_x_at0.0477 Chemokine (C-X-C motif) ligand 5

**Gene Probe** **p-Value** **Description**

219667_s_at 0.000968 B-cell scaffold protein with ankyrin repeats 1

205049_s_at0.00113CD79A antigen

219497_s_at0.00132B-cell CLL/lymphoma 11A (zinc finger protein)

221239_s_at0.00132SH2 domain cont. phosphatase anchor prtn 1

235401_s_at0.00179Fc receptor homolog expressed in B cells

230983_at 0.00207B-cell novel protein 1

205671_s_at 0.0024MHC, class II, DO beta

228599_at 0.00278Membrane-spanning 4-domains, subfamily A

220068_at 0.00278Pre-B lymphocyte gene 3

235982_at 0.0032Fc receptor-like protein 1

226384_at 0.0032HTPAP protein

243968_x_at0.0032tn64g01.x1 NCI_CGAP_Lym12

39318_at0.00369T-cell leukemia/lymphoma 1A

213891_s_at0.00369Transcription factor 4

206126_at 0.00424Burkitt lymphoma receptor 1

206398_s_at0.00424CD19 antigen

205861_at 0.00424Spi-B transcription factor (Spi-1/PU.1 related)

207655_s_at0.00487B-cell linker

228592_at 0.00487Membrane-spanning 4-domains, subfamily A

209995_s_at0.00487T-cell leukemia/lymphoma 1A

209583_s_at0.00638CD200 antigen

38521_at0.00638CD22 antigen

218781_at 0.00638SMC6 structural maintenance of chromosomes

215925_s_at0.00728CD72 antigen

212827_at 0.00728Immunoglobulin heavy constant mu

227198_at 0.00728Lymphoid nuclear protein related to AF4

203221_at 0.00728Transducin-like enhancer of split 1

227533_at 0.00728zg78d04.s1 Soares_fetal_heart_NbHH19W

206759_at 0.00944Fc fragment of IgE, low affinity II

217418_x_at0.00944Membrane-spanning 4-domains, subfamily A

206255_at 0.0122B lymphoid tyrosine kinase

44790_s_at 0.0122Chromosome 13 open reading frame 18

213674_x_at0.0155Immunoglobulin heavy constant delta

219073_s_at0.0155Oxysterol binding protein-like 10

227646_at 0.0175602508050F1 NIH_MGC_79 Homo sapiens

210356_x_at0.0175Membrane-spanning 4-domains, subfamily A

205297_s_at0.0197CD79B antigen

219471_at 0.0197Chromosome 13 open reading frame 18

226912_at 0.0197Zinc finger, DHHC domain containing 23

227224_at 0.0222Angiopoietin-like 1

235372_at 0.0222Fc receptor homolog expressed in B cells

210448_s_at0.0222Purinergic receptor P2X

207777_s_at0.0222SP140 nuclear body protein

229513_at0.0222Spermatid perinuclear RNA binding protein

226122_at0.0222

243780_at0.0249CDNA FLJ46553 fis, clone THYMU3038879

222891_s_at0.0278B-cell CLL/lymphoma 11A (zinc finger protein)

220059_at0.0311BCR downstream signaling 1

230877_at0.0347Immunoglobulin heavy constant delta

219498_s_at0.0387B-cell CLL/lymphoma 11A (zinc finger protein)

206478_at.0387KIAA0125

201689_s_at0.043Tumor protein D52

222915_s_at0.0477B-cell scaffold protein with ankyrin repeats 1

203642_s_at0.0477COBL-like 1

225081_s_at0.0477Transcription factor RAM2


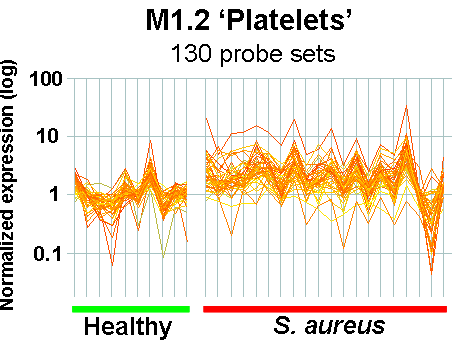

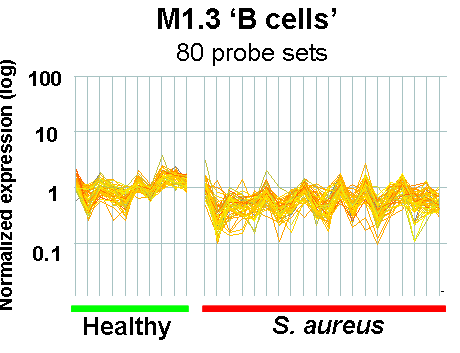

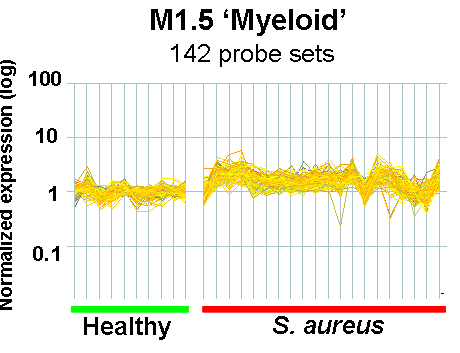

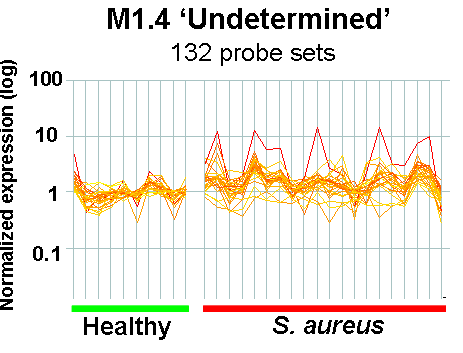


**Gene Probe** **p-Value** **Description**

224783_at0.0006Hypothetical protein MGC29814

211999_at0.0024H3 histone, family 3B (H3.3B)

227718_at0.0032Purine-rich element binding protein B

202558_s_at0.0032Stress 70 protein chaperone

205214_at0.00424Serine/threonine kinase 17b (apoptosis-inducing)

41577_at0.00424Protein phosphatase 1, regulatory (inhibitor) subunit 16B

201356_at0.00487601810961R1 NIH_MGC_48 Homo sapiens cDNA 208881_x_at0.00728Isopentenyl-diphosphate delta isomerase

210281_s_at0.00728Zinc finger protein 198

36711_at0.00728

204615_x_at0.0107Isopentenyl-diphosphate delta isomerase

208632_at0.0155Ring finger protein 10

226650_at0.0197Hypothetical protein LOC90637

215948_x_at0.0222Zinc finger protein 237

238509_at0.0222Cullin 1

202776_at0.0222Estrogen receptor binding protein

207332_s_at0.0249Transferrin receptor (p90, CD71)

226370_at0.0347Kelch-like 15 (Drosophila)

233952_s_at0.0347Zinc finger protein 295

200779_at0.0347Activating transcription factor 4

223296_at0.0387Mitochondrial carrier protein

241924_at0.043Methyl CpG binding protein 2 (Rett syndrome)

212665_at0.043TCDD-inducible poly(ADP-ribose) polymerase

229106_at0.043Dynein light chain 2

208720_s_at0.043RNA-binding region (RNP1, RRM) containing 2

222669_s_at0.0477Shwachman-Bodian-Diamond syndrome

**Gene Probe** **p-Value** **Description**

200743_s_at0.000219Tripeptidyl peptidase I

215049_x_at0.000366CD163 antigen

200765_x_at0.000366Catenin (cadherin-associated protein), alpha 1, 102kDa

218454_at0.000432Hypothetical protein FLJ22662

207677_s_at0.00051Neutrophil cytosolic factor 4, 40kDa

203773_x_at0.00051Biliverdin reductase A

217764_s_at0.0006RAB31, member RAS oncogene family

202833_s_at0.0006Serine (or cysteine) proteinase inhibitor, clade A

221731_x_at0.000705Chondroitin sulfate proteoglycan 2 (versican)

227276_at0.000705Plexin domain containing 2

223703_at0.000827Chromosome 10 open reading frame 11

204971_at0.000827Cystatin A (stefin A)

211729_x_at0.000827Biliverdin reductase A

213503_x_at0.000968Transcribed locus, strongly similar to annexin

236297_at0.00113Plexin domain containing 2

203501_at0.00113aminopeptidase

212334_at0.00113Glucosamine (N-acetyl)-6-sulfatase

200838_at0.00113Cathepsin B

209949_at0.00113Neutrophil cytosolic factor 2

200660_at0.00132S100 calcium binding protein A11 (calgizzarin)

206420_at0.00154Immunoglobulin superfamily, member 6

204619_s_at0.00154Chondroitin sulfate proteoglycan 2 (versican)

208702_x_at0.00179Amyloid beta (A4) precursor-like protein 2

217865_at0.00207Ring finger protein 130

212737_at0.00207Chorionic somatomammotropin hormone 2

213095_x_at0.00278Allograft inflammatory factor 1

201536_at0.00278Dual specificity phosphatase 3

215051_x_at0.00278Allograft inflammatory factor 1

211284_s_at0.00278Granulin

218217_at0.00278Serine carboxypeptidase 1

217989_at0.00278Dehydrogenase/reductase (SDR family) member 8

210784_x_at0.0032Leukocyte immunoglobulin-like receptor

211135_x_at0.0032Leukocyte immunoglobulin-like receptor

223501_at0.00369xe73e12.x1 NCI_CGAP_Ut3 Homo sapiens cDNA

203561_at0.00369Fc fragment of IgG, low affinity IIa, receptor for (CD32)

201743_at0.00424CD14 antigen

213716_s_at0.00424Secreted and transmembrane 1

218610_s_at0.00487Hypothetical protein FLJ11151

208146_s_at0.00487Carboxypeptidase, vitellogenic-like

202902_s_at0.00487Cathepsin S

208982_at0.00558UI-HF-BK0-aab-h-05-0-UI.s1 NIH_MGC_36

209901_x_at0.00558Allograft inflammatory factor 1

219259_at0.00558Sema domain, immunoglobulin domain (Ig)

205603_s_at0.00558Diaphanous homolog 2 (Drosophila)

207857_at0.00638Leukocyte immunoglobulin-like receptor, subfamily A

204924_at0.00638Toll-like receptor 2

221802_s_at0.00728KIAA1598

226728_at0.0083Solute carrier family 27 (fatty acid transporter), member 1

212993_at0.0083MRNA; cDNA DKFZp667B1718

224374_s_at0.0083Elastin microfibril interfacer 2

223405_at0.00944N-acetylneuraminate pyruvate lyase

210895_s_at0.00944CD86 antigen (CD28 antigen ligand 2, B7-2 antigen)

219358_s_at0.0107Centaurin, alpha 2

202943_s_at0.0138N-acetylgalactosaminidase, alpha-

210844_x_at0.0138Catenin

218240_at0.0138NFKB inhibitor interacting Ras-like 2

217118_s_at0.0155Chromosome 22 open reading frame 9

202367_at0.0155Cut-like 1, CCAAT displacement protein

222218_s_at0.0175Paired immunoglobin-like type 2 receptor alpha

204393_s_at0.0175Acid phosphatase, prostate

202030_at0.0175Branched chain ketoacid dehydrogenase kinase

205237_at0.0197Ficolin (collagen/fibrinogen domain containing) 1

208018_s_at0.0197Hemopoietic cell kinase

202878_s_at0.0197Complement component 1, q subcomponent, receptor 1

207697_x_at0.0197Leukocyte immunoglobulin-like receptor, subfamily B

208130_s_at0.0222Thromboxane A synthase 1

216041_x_at0.0222Granulin

202795_x_at0.0249Tara-like protein

204588_s_at0.0249Solute carrier family 7

200678_x_at0.0311Granulin

209263_x_at0.0311Transmembrane 4 superfamily member 7

205859_at0.0347Lymphocyte antigen 86

208981_at0.0347UI-HF-BK0-aab-h-05-0-UI.s1 NIH_MGC_36 Homo sapiens cDNA

205686_s_at0.0347CD86 antigen

211336_x_at0.0347Leukocyte immunoglobulin-like receptor, subfamily B

213275_x_at0.0387601487244F1 NIH_MGC_69 Homo sapiens cDNA

207104_x_at0.0387Leukocyte immunoglobulin-like receptor, subfamily B

58780_s_at0.0387Hypothetical protein FLJ10357

203508_at0.043Tumor necrosis factor receptor superfamily, member 1B

208890_s_at0.0477Plexin B2

205936_s_at0.0477Hexokinase 3 (white cell)

212820_at0.0477Dmx-like 2

200714_x_at0.0477Amplified in osteosarcoma


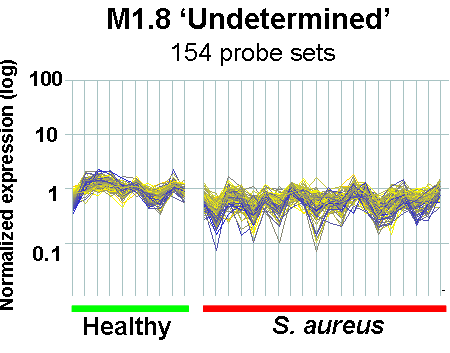

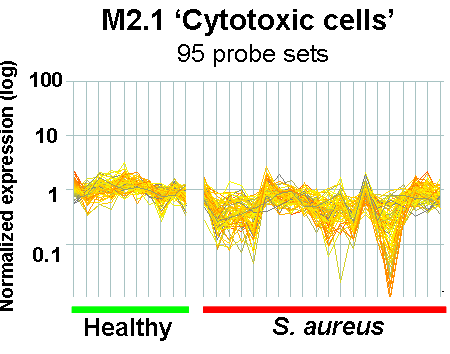


**Gene Probe** **p-Value** **Description**

219130_at 2.92e-5 Hypothetical protein FLJ10287

231844_at 3.54e-5 Hypothetical protein MGC27345

64418_at 9.02e-5 CDNA FLJ34482 fis, clone HLUNG2004067

227026_at 9.02e-5 Transcribed locus

212453_at 0.000108 KIAA1279

230142_s_at 0.000129 Cold inducible RNA binding protein

201447_at 0.000129 TIA1 cytotoxic granule-associated RNA binding protein

228029_at 0.000155 KIAA1982 protein

204882_at 0.000184 Rho GTPase activating protein 25

219128_at 0.000219 Hypothetical protein FLJ20558

209828_s_at 0.000219 Interleukin 16

222028_at 0.000261 Zinc finger protein 45

203694_s_at 0.000309 DEAH (Asp-Glu-Ala-His) box polypeptide 16

204676_at 0.000366 DKFZP564K2062 protein

209497_s_at 0.000366 RNA binding motif protein 30

238653_at 0.000432 Transcribed locus

225816_at 0.000432 PHD finger protein 17

221865_at 0.00051 Chromosome 9 open reading frame 91

219303_at 0.00051 Chromosome 13 open reading frame 7

218104_at 0.0006 Testis expressed sequence 10

203159_at 0.0006 Glutaminase

203427_at 0.0006 ASF1 anti-silencing function 1 homolog A

41329_at 0.0006 Ezrin-binding partner PACE-1

213838_at 0.000705 RAN binding protein 9

228041_at 0.000827 2-aminoadipic 6-semialdehyde dehydrogenase

226109_at 0.000968 Chromosome 21 open reading frame 91

235612_at 0.000968 Transcribed locus

228916_at 0.000968 CWF19-like 2, cell cycle control

227626_at 0.000968 Chromosome 6 open reading frame 33

210541_s_at 0.00113 Ret finger protein

228920_at 0.00113 Hypothetical protein LOC339324

228336_at 0.00132 KIAA1935 protein

224430_s_at 0.00132 Mitochondrial translation optimization 1 homolog

229367_s_at 0.00132 GTPase, IMAP family member 6

201270_x_at 0.00132 KIAA1068 protein

203614_at 0.00154 Similar to hypothetical protein B230397C21

204327_s_at 0.00154 Zinc finger protein 202

218371_s_at 0.00154 Paraspeckle component 1

213626_at 0.00154 Carbonic reductase 4

214129_at 0.00166 Phosphodiesterase 4D interacting protein

223297_at 0.00207 Hypothetical protein MGC4268

200854_at 0.00207 Nuclear receptor co-repressor 1

228963_at 0.00207 7f86e10.x1 NCI_CGAP_Pr28

226020_s_at 0.0024 OMA1 homolog, zinc metallopeptidase

226371_at 0.0024 Jumonji, AT rich interactive domain 1A

202060_at 0.0024 SH2 domain binding protein 1

203143_s_at 0.0024 T cell receptor alpha chain

222811_at 0.00278 Hypothetical protein FLJ11171

226604_at 0.00278 SMILE protein

219123_at 0.0032 Zinc finger protein 232

207338_s_at 0.0032 Zinc finger protein 200

218614_at 0.0032 Hypothetical protein FLJ20696

218626_at 0.00369 Eukaryotic translation initiation factor 4E nuclear import factor 1

218968_s_at 0.00369 Zinc finger protein 64 homolog

210053_at 0.00369 TAF5 RNA polymerase II

212222_at 0.00424 Proteasome (prosome, macropain) activator subunit 4

226180_at 0.00487 nab65f06.x1

202184_s_at 0.00487 Nucleoporin 133kDa

235215_at 0.00487 Transcribed locus

216221_s_at 0.00487 Vacuolar protein sorting 35 (yeast)

219467_at 0.00487 Hypothetical protein FLJ20125

227335_at 0.00558 Chromosome 20 open reading frame 158

218716_x_at 0.00638 Mitochondrial translation optimization 1 homolog

214988_s_at 0.00638 SON DNA binding protein

227426_at 0.00638 Son of sevenless homolog 1

228071_at 0.00728 GTPase, IMAP family member 7

228167_at 0.00728 Kelch-like 6 (Drosophila)

223351_at 0.00728 yx46f01.s1 Soares melanocyte 2NbHM

226821_at 0.0083 yf85g08.s1 Soares infant brain 1NIB

221081_s_at 0.0083 Hypothetical protein FLJ22457

208121_s_at 0.00944 Protein tyrosine phosphatase, receptor type, O

206734_at 0.00944 Jerky homolog-like (mouse)

207513_s_at 0.00944 Zinc finger protein 189

226230_at 0.00944 KIAA1387 protein

225370_at 0.00944 UI-H-BI2-aga-c-07-0-UI.s1 NCI_CGAP_Sub4 Homo sapiens cDNA clone

202453_s_at 0.00944 General transcription factor IIH, polypeptide 1, 62kDa

219777_at 0.0107 GTPase, IMAP family member 6

226031_at 0.0107 Hypothetical protein FLJ20097

228453_at 0.0107 Hypothetical protein LOC284267

236401_at 0.0122 nf61d03.s1 NCI_CGAP_Co3 Homo sapiens cDNA clone IMAGE:924389 3', mRNA sequence.

228805_at 0.0122 FLJ44216 protein

216863_s_at 0.0146

213405_at 0.0155 RAB22A, member RAS oncogene family

226242_at 0.0155 Hypothetical protein DKFZp547B1713

226032_at 0.0175 Caspase 2

229969_at 0.0197 AV723931 HTB Homo sapiens cDNA clone HTBAQD04 5', mRNA sequence.

218138_at 0.0197 McKusick-Kaufman syndrome

226958_s_at 0.0197 Similar to HSPC296

212116_at 0.0197 Ret finger protein

218242_s_at 0.0222 Suppressor of variegation 4-20 homolog 1

202983_at 0.0222 SWI/SNF related, matrix associated, actin dependent regulator of chromatin, subfamily a, member 3

203611_at 0.0249 Telomeric repeat binding factor 2

209662_at 0.0278 Centrin, EF-hand protein, 3 (CDC31 homolog, yeast)

225290_at 0.0311 CDNA clone IMAGE:5261903, partial cds

203584_at 0.0311 KIAA0103

212740_at 0.0311 Phosphoinositide-3-kinase, regulatory subunit 4, p150

224959_at 0.0347 Solute carrier family 26 (sulfate transporter), member 2

225988_at 0.0347 Hect domain and RLD 4

226867_at 0.0347 Chromosome 9 open reading frame 55

212632_at 0.0387 Syntaxin 7

218455_at 0.043 NFS1 nitrogen fixation 1

233665_at 0.043 Mitochondrial translation optimization 1 homolog

227102_at 0.0477 Tripartite motif-containing 35

235152_at 0.0477 Homo sapiens, clone IMAGE:5218412, mRNA

**Gene Probe** **p-Value** **Description**

205232_s_at 1.98e-5 Platelet-activating factor acetylhydrolase 2, 40kDa

218638_s_at 7.51e-5 Spondin 2, extracellular matrix protein

207734_at 0.000108 Hypothetical protein FLJ20340

225525_at 0.000108 KIAA1671 protein

206267_s_at 0.000108 Megakaryocyte-associated tyrosine kinase

220684_at 0.000129 T-box 21

212070_at 0.000129 G protein-coupled receptor 56

205821_at 0.000155 Killer cell lectin-like receptor subfamily K, member 1

204960_at 0.000155 Protein tyrosine phosphatase, receptor type, C-associated

207351_s_at 0.000155 SH2 domain protein 2A

236782_at 0.000184 Sterile alpha motif domain containing 3

210606_x_at 0.000184 Killer cell lectin-like receptor subfamily D, member 1

47069_at 0.000219 Rho GTPase activating protein 8

230753_at 0.000219 Similar to RIKEN cDNA 4930424G05

206118_at 0.000261 Signal transducer and activator of transcription 4

234165_at 0.000366 Prostaglandin D2 receptor (DP)

205831_at 0.000366 CD2 antigen (p50), sheep red blood cell receptor

236265_at 0.000432 Homo sapiens, clone IMAGE:3887266, mRNA

213830_at 0.00051 T-cell receptor rearranged delta-chain mRNA V-region

216191_s_at 0.00051 T-cell receptor rearranged delta-chain mRNA V-region

207795_s_at 0.000827 Killer cell lectin-like receptor subfamily D, member 1

225688_s_at 0.000968 Pleckstrin homology-like domain, family B, member 2

226625_at 0.00113 Transforming growth factor, beta receptor III

213906_at 0.00113 V-myb myeloblastosis viral oncogene homolog

210972_x_at 0.00179 T-cell receptor rearranged alpha-chain V-region (V-D-J)

210006_at 0.00179 DKFZP564O243 protein

235919_at 0.00207 Transcribed locus

204160_s_at 0.00207 Ectonucleotide pyrophosphatase/phosphodiesterase 4

219529_at 0.00207 Chloride intracellular channel 3

217143_s_at 0.00207 T cell receptor delta locus

210164_at 0.0024 Granzyme B

204070_at 0.0024 Retinoic acid receptor responder (tazarotene induced) 3

211685_s_at 0.0024 Neurocalcin delta

215894_at 0.00278 Prostaglandin D2 receptor (DP)

231776_at 0.00278 Eomesodermin homolog (Xenopus laevis)

218764_at 0.0032

210763_x_at 0.0032 Natural cytotoxicity triggering receptor 3

214617_at 0.00369 tj08g03.x1 NCI_CGAP_Gas4.

223836_at 0.00424 Ksp37 protein

232914_s_at 0.00424 Synaptotagmin-like 2

230464_at 0.00487 Endothelial differentiation, sphingolipid G-ptn-coupled rec, 8

228774_at 0.00487 Chromosome 9 open reading frame 81

215332_s_at 0.00487 CD8 antigen, beta polypeptide 1 (p37)

221267_s_at 0.00558 Chromosome 19 open reading frame 27

202761_s_at 0.00558 Spectrin repeat containing, nuclear envelope 2

211144_x_at 0.00558 Similar to T-cell receptor gamma chain V

215806_x_at 0.00558 Similar to T-cell receptor gamma chain V

205495_s_at 0.00638 Granulysin

210288_at 0.00638 Killer cell lectin-like receptor subfamily G, member 1

203713_s_at 0.00638 Lethal giant larvae homolog 2 (Drosophila)

220646_s_at 0.00728 Killer cell lectin-like receptor subfamily F, member 1

207979_s_at 0.0083 CD8 antigen, beta polypeptide 1 (p37)

207840_at 0.0083 CD160 antigen

219541_at 0.00944 Hypothetical protein FLJ20406

209813_x_at 0.00944 T cell receptor gamma variable 9

202931_x_at 0.00944 Bridging integrator 1

219304_s_at 0.0107 DNA-damage inducible protein 1

204731_at 0.0122 Transforming growth factor, beta receptor III

205291_at 0.0138 Interleukin 2 receptor, beta

205171_at 0.0155 Protein tyrosine phosphatase, non-receptor type 4

209883_at 0.0155 Glycosyltransferase 25 domain containing 2

231241_at 0.0175 Schlafen 5

241803_s_at 0.0175 (clone tec232) mRNA.

216920_s_at 0.0197 Similar to T-cell receptor gamma chain V region PT-gamma-1/2 precursor

37145_at 0.0197 Homo sapiens NKG5 gene, complete cds.

207723_s_at 0.0222 Killer cell lectin-like receptor subfamily C, member 2

214450_at 0.0222 Cathepsin W (lymphopain)

212599_at 0.0222 Autism susceptibility candidate 2

206785_s_at 0.0278 Killer cell lectin-like receptor subfamily C, member 2

213658_at 0.0311 Hypothetical protein DKFZp547K1113

203562_at 0.0311 Fasciculation and elongation protein zeta 1 (zygin I)

214567_s_at 0.0387 Chemokine (C motif) ligand 2

214995_s_at 0.043 KIAA0907 protein

213915_at 0.043 Natural killer cell group 7 sequence

205488_at 0.0477 Granzyme A

225706_at 0.0477 Homo sapiens islet cell autoantigen 1

211597_s_at 0.0477 Homeodomain-only protein

223259_at 0.0477 ORM1-like 3


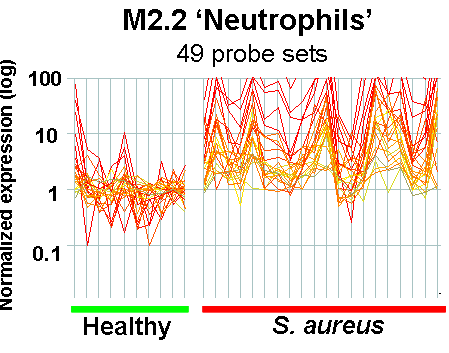


**Gene Probe** **p-Value** **Description**

202018_s_at 2.92e-5Lactotransferrin

231688_at 2.92e-5Transcribed locus

206697_s_at 3.54e-5Haptoglobin

205033_s_at 4.29e-5Defensin, alpha 1, myeloid-related sequence

206676_at 5.18e-5Carcinoembryonic antigen-related cell adhesion molecule 8

204351_at 5.18e-5S100 calcium binding protein P

207269_at 7.51e-5Defensin, alpha 4, corticostatin

212531_at 9.02e-5Lipocalin 2 (oncogene 24p3)

202252_at 9.02e-5RAB13, member RAS oncogene family

220570_at 0.000108Resistin

205557_at 0.000129Bactericidal/permeability-increasing protein

201161_s_at 0.000261Cold shock domain protein A

212768_s_at 0.000309

206177_s_at 0.000366Arginase, liver

206834_at 0.000432Hemoglobin, delta

209369_at 0.000705Annexin A3

213515_x_at 0.000827Hemoglobin, gamma G

201160_s_at 0.00179Cold shock domain protein A

211657_at 0.0024

210254_at 0.00278Membrane-spanning 4-domains, subfamily A, member 3

224889_at 0.00487Forkhead box O3A

203936_s_at 0.00558Matrix metalloproteinase 9

205513_at 0.00728Transcobalamin I

209211_at 0.0155Kruppel-like factor 5


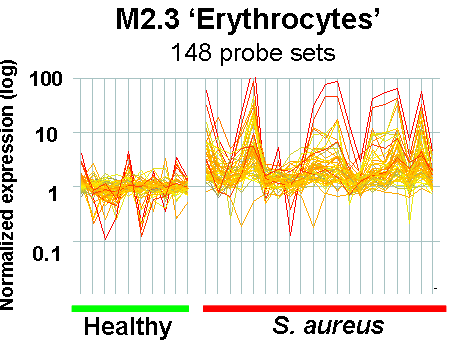


**Gene Probe** **p-Value** **Description**

204466_s_at 1.62e-5 Synuclein, alpha

231078_at 4.29e-5 Mitochondrial solute carrier protein

226179_at 6.24e-5 FP15737

221920_s_at 6.24e-5 Mitochondrial solute carrier protein

203966_s_at 9.02e-5 Protein phosphatase 1A (formerly 2C)

207827_x_at 0.000155 Synuclein, alpha (non A4 component of amyloid precursor)

202974_at 0.000155 Membrane protein, palmitoylated 1, 55kDa

219672_at 0.000155 Erythroid associated factor

204467_s_at 0.000261 Synuclein, alpha (non A4 component of amyloid precursor)

235095_at 0.000261 Hypothetical LOC146439

204505_s_at 0.000309 Erythrocyte membrane protein band 4.9 (dematin)

217748_at 0.000366 Adiponectin receptor 1

222528_s_at 0.000366 Mitochondrial solute carrier protein

223266_at 0.000366 Amyotrophic lateral sclerosis 2 (juvenile) chromosome region

205950_s_at 0.000432 Carbonic anhydrase I

215438_x_at 0.00051 G1 to S phase transition 1

242335_at 0.00051 EST95296 Activated T-cells II Homo sapiens cDNA

231274_s_at 0.000705 Mitochondrial solute carrier protein

204848_x_at 0.000705 Hemoglobin, gamma A

237819_at 0.000827 CAMP responsive element binding protein 3-like 2

202130_at 0.000968 RIO kinase 3 (yeast)

211546_x_at 0.00113 Synuclein, alpha (non A4 component of amyloid precursor)

206302_s_at 0.00113 Nudix (nucleoside diphosphate linked moiety X)-type motif 4

222529_at 0.00113 Mitochondrial solute carrier protein

223012_at 0.00113 UBX domain containing 1

221932_s_at 0.00113 Chromosome 14 open reading frame 87

204131_s_at 0.00132 Forkhead box O3A

211699_x_at 0.00154 Hemoglobin, alpha 1

204419_x_at 0.00154 Hemoglobin, gamma G

209845_at 0.00207 Makorin, ring finger protein, 1

224690_at 0.0024 Chromosome 20 open reading frame 108

228361_at 0.00278 E2F transcription factor 2

221675_s_at 0.0032 Choline phosphotransferase 1

222730_s_at 0.0032 Zinc finger, DHHC domain containing 2

211475_s_at 0.00369 BCL2-associated athanogene

202468_s_at 0.00487 Catenin (cadherin-associated protein), alpha-like 1

202364_at 0.00558 MAX interactor 1

218136_s_at 0.00558 Mitochondrial solute carrier protein

220466_at 0.00558 Hypothetical protein FLJ13215

209018_s_at 0.00638 PTEN induced putative kinase 1

213843_x_at 0.00638 Solute carrier family 6

201178_at 0.00728 F-box protein 7

202387_at 0.0083 BCL2-associated athanogene

204187_at 0.0083 Guanosine monophosphate reductase

224693_at 0.0122 Chromosome 20 open reading frame 108

200075_s_at 0.0122 Guanylate kinase 1

215499_at 0.0138 Mitogen-activated protein kinase kinase 3

226009_at 0.0138 Deleted in a mouse model of primary ciliary dyskinesia

212540_at 0.0155 Cell division cycle 34

207801_s_at 0.0175 Ring finger protein 10

212512_s_at 0.0197 Coactivator-associated arginine methyltransferase 1

205570_at 0.0197 Phosphatidylinositol-4-phosphate 5-kinase, type II, alpha

226811_at 0.0249 Family with sequence similarity 46, member C

237163_x_at 0.0249 Similar to protein phosphatase 2A 48 kDa regulatory subunit isoform 1

207667_s_at 0.0311 Mitogen-activated protein kinase kinase 3

215684_s_at 0.0387 Homo sapiens mRNA; cDNA DKFZp586O0223


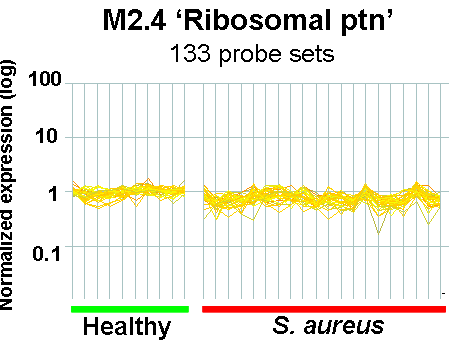


**Gene Probe** **p-Value** **Description**

208117_s_at0.000309LAS1-like

201230_s_at0.000827Ariadne homolog 2

203113_s_at0.000968Eukaryotic translation elongation factor 1 delta

212197_x_at0.000968Myosin phosphatase-Rho interacting protein

217906_at0.00113Kelch domain containing 2

204772_s_at0.00179Transcription termination factor, RNA polymerase I

202408_s_at0.00207PRP31 pre-mRNA processing factor 31 homolog

218421_at0.00207Ceramide kinase

203818_s_at0.00424Splicing factor 3a, subunit 3, 60kDa

212114_at0.00487Similar to microtubule-associated proteins 1A/1B light chain 3

212017_at0.00558Hypothetical protein LOC130074

221519_at0.00638Split hand/foot malformation (ectrodactyly) type 3

208826_x_at0.00638Histidine triad nucleotide binding protein 1

201272_at0.00638Aldo-keto reductase family 1, member B1

222099_s_at0.00728Chromosome 19 open reading frame 13

210825_s_at0.00944predicted protein of HQ0720

220755_s_at0.0107Chromosome 6 open reading frame 48

212995_x_at0.0155Hypothetical protein FLJ14346

217988_at0.0155Cyclin B1 interacting protein 1

214042_s_at0.0175Myelodysplasia syndrome 1

213846_at0.0197Cytochrome c oxidase subunit VIIc

200631_s_at0.0197SET translocation

217256_x_at0.0249

214317_x_at0.0278Ribosomal protein S9

221726_at0.0311Ribosomal protein L22

202365_at0.0311Hypothetical protein MGC5139

209538_at0.0347Zinc finger protein 32 (KOX 30)

234875_at0.0387Homo sapiens rpL7a pseudogene, clone 3a.

219817_at0.0387Apoptosis-related protein PNAS-1

217969_at0.043Chromosome 11 open reading frame2

213166_x_at0.043CDNA clone MGC:8772 IMAGE:3862861, complete cds

221494_x_at0.0477Eukaryotic translation initiation factor 3, subunit 12

210792_x_at0.0477CD27-binding (Siva) protein


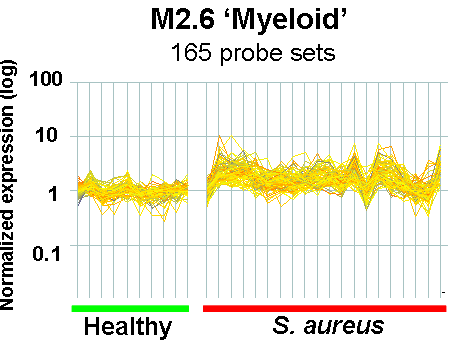


**Gene Probe** **p-Value** **Description**

208949_s_at3.54e-5Lectin, galactoside-binding, soluble, 3

201200_at5.18e-5Cellular repressor of E1A-stimulated genes 1

200958_s_at6.24e-5Syndecan binding protein

203041_s_at7.51e-5Lysosomal-associated membrane protein 2

223392_s_at0.000129Zinc finger protein 537

202201_at0.000219Biliverdin reductase B

229770_at0.000261Hypothetical protein FLJ31978

203167_at0.000309Tissue inhibitor of metalloproteinase 2

203535_at0.000309S100 calcium binding protein A9 (calgranulin B)

205627_at0.000309Cytidine deaminase

204446_s_at0.00051Arachidonate 5-lipoxygenase

222934_s_at0.0006C-type lectin, superfamily member 9

209179_s_at0.000705Leukocyte receptor cluster (LRC) member 4

202897_at0.000827Protein tyrosine phosphatase

217762_s_at0.000827RAB31, member RAS oncogene family

205068_s_at0.000968Rho GTPase activating protein 26

226066_at0.000968Microphthalmia-associated transcription factor

205119_s_at0.000968Formyl peptide receptor 1

222105_s_at0.00113NFKB inhibitor interacting Ras-like 2

221541_at0.00113Hypothetical protein DKFZp434B044

203645_s_at0.00113CD163 antigen

218773_s_at0.00113Methionine sulfoxide reductase B2

211404_s_at0.00113Amyloid beta (A4) precursor-like protein 2

235593_at0.00113Zinc finger homeobox 1b

226026_at0.00132Disrupted in renal carcinoma 2

228325_at0.00154KIAA0146 protein

202192_s_at0.00179

204232_at0.00179Fc fragment of IgE

200839_s_at0.00207Cathepsin B

202917_s_at0.00207S100 calcium binding protein A8 (calgranulin A)

223502_s_at0.00207Tumor necrosis factor (ligand) superfamily, member 13b

228499_at0.002076-phosphofructo-2-kinase/fructose-2,6-biphosphatase 4

211429_s_at0.00207Serine (or cysteine) proteinase inhibitor, clade A

201422_at0.0024Interferon, gamma-inducible protein 30

224560_at0.00278Tissue inhibitor of metalloproteinase 2

218627_at0.00278Hypothetical protein FLJ11259

231579_s_at0.00278Tissue inhibitor of metalloproteinase 2

210225_x_at0.00278Leukocyte immunoglobulin-like receptor, subfamily B

202803_s_at0.0032Integrin, beta 2

205568_at0.0032Aquaporin 9

213187_x_at0.0032602567289F1 NIH_MGC_77

222689_at0.0032Phytoceramidase, alkaline

225685_at0.00369CDC42 effector protein (Rho GTPase binding) 3

203814_s_at0.00369NAD(P)H dehydrogenase, quinone 2

202426_s_at0.00396Retinoid X receptor, alpha

200677_at0.00424Pituitary tumor-transforming 1 interacting protein

212506_at0.00424Phosphatidylinositol binding clathrin assembly protein

211576_s_at0.00487Solute carrier family 19 (folate transporter), member 1

225373_at0.00487Full-length cDNA clone CS0DI015YJ11

224573_at0.00487Similar to DNA segment, Chr 11

205922_at0.00558Vanin 2

226577_at0.00558Presenilin 1 (Alzheimer disease 3)

211133_x_at0.00638Leukocyte immunoglobulin-like receptor, subfamily B

211067_s_at0.00638Growth arrest-specific 7

209286_at0.00728CDC42 effector protein (Rho GTPase binding) 3

223280_x_at0.0083Membrane-spanning 4-domains, subfamily A, member 6A

227367_at0.00944Solute carrier organic anion transporter family, 3A1

213006_at0.00944KIAA0146 protein

213702_x_at0.00944N-acylsphingosine amidohydrolase (acid ceramidase) 1

201642_at0.0107Interferon gamma receptor 2 (IFN gamma transducer 1)

212041_at0.0122ATPase, H+ transporting, lysosomal 38kDa

205640_at0.0122Aldehyde dehydrogenase 3 family, member B1

204204_at0.0138Solute carrier family 31 (copper transporters), member 2

214875_x_at0.0155Amyloid beta (A4) precursor-like protein 2

234942_s_at0.0155Thyroid hormone receptor associated protein 3

224652_at0.0175Chromosome 10 open reading frame 9

221036_s_at0.0175Anterior pharynx defective 1B-like

223553_s_at0.0175Docking protein 3

204053_x_at0.0197Phosphatase and tensin homolog

241742_at0.0197PRAM-1 protein

217931_at0.0222Trinucleotide repeat containing 5

208540_x_at0.0249

223303_at0.0249UNC-112 related protein 2

207571_x_at0.0249Chromosome 1 open reading frame 38

202100_at0.0249V-ral simian leukemia viral oncogene homolog B

214084_x_at0.0278General transcription factor II, i, pseudogene 1

227013_at0.0278LATS, large tumor suppressor, homolog 2

210340_s_at0.0311Colony stimulating factor 2 receptor, alpha, low-affinity (granulocyte-macrophage)

204122_at0.0311TYRO protein tyrosine kinase binding protein

223519_at0.0311cr43e01.x1 Human bone marrow stromal cells

201336_at0.0347Vesicle-associated membrane protein 3

204961_s_at0.0347Neutrophil cytosolic factor 1

207765_s_at0.0387KIAA1539

210422_x_at0.0387Solute carrier family 11, member 1

229101_at0.043Hypothetical protein LOC150166

227929_at0.0477Homo sapiens, clone IMAGE:5277945, mRNA

203005_at0.0477Lymphotoxin beta receptor (TNFR superfamily, member 3)


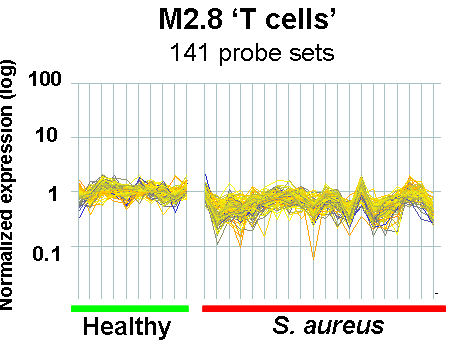


**Gene Probe** **p-Value** **Description**

219315_s_at2.92e-5Chromosome 16 open reading frame 30

212641_at5.18e-5

215967_s_at5.18e-5Lymphocyte antigen 9

218918_at7.51e-5Mannosidase, alpha, class 1C, member 1

212313_at0.000108Hypothetical protein MGC29816

210202_s_at0.000108Bridging integrator 1

57082_at0.000155LDL receptor adaptor protein

213340_s_at0.000219KIAA0495

202969_at0.000219Dual-specificity tyrosine-(Y)-phosphorylation regulated kinase 2

221601_s_at0.000309Regulator of Fas-induced apoptosis

205790_at0.000366Src family associated phosphoprotein 1

219700_at0.000366Plexin domain containing 1

47560_at0.000366Latrophilin 1

216262_s_at0.00051

212400_at0.0006Early estrogen-induced gene 1 protein

239122_at0.0006Regulator of Fas-induced apoptosis

218437_s_at0.000705Leucine zipper transcription factor-like 1

219528_s_at0.000705B-cell CLL/lymphoma 11B (zinc finger protein)

212642_s_at0.000705

214177_s_at0.000827Pre-B-cell leukemia transcription factor interacting protein 1

214081_at0.000827Plexin domain containing 1

219734_at0.000968SID1 transmembrane family, member 1

203386_at0.000968TBC1 domain family, member 4

225864_at0.000968Breast cancer membrane protein 101

228046_at0.000968Hypothetical protein LOC152485

206761_at0.00113CD96 antigen

218517_at0.00113PHD finger protein 17

207339_s_at0.00113Lymphotoxin beta (TNF superfamily, member 3)

227686_at0.00132Hypothetical protein BC008322

206337_at0.00132Chemokine (C-C motif) receptor 7

202968_s_at0.00132Dual-specificity tyrosine-(Y)-phosphorylation reg kinase 2

220054_at0.00154Interleukin 23, alpha subunit p19

213971_s_at0.00179Similar to KIAA0160 gene product is novel

236826_at0.00207

209604_s_at0.00207GATA binding protein 3

213039_at0.00207Rho/rac guanine nucleotide exchange factor (GEF) 18

203413_at0.00207NEL-like 2 (chicken)

219922_s_at0.00207Latent transforming growth factor beta binding protein 3

221602_s_at0.00207Regulator of Fas-induced apoptosis

204951_at0.0024Ras homolog gene family, member H

201313_at0.0024Enolase 2 (gamma, neuronal)

203385_at0.0024Diacylglycerol kinase, alpha 80kDa

222696_at0.00278Axin 2 (conductin, axil)

218532_s_at0.00278Hypothetical protein FLJ20152

222307_at0.00278we49g06.x1 NCI_CGAP_Co3 Homo sapiens

210915_x_at0.00278T-cell receptor rearranged beta-chain V-region

212771_at0.0032Chromosome 10 open reading frame 38

213534_s_at0.00424PAS domain containing serine/threonine kinase

218932_at0.00424Hypothetical protein FLJ20729

213193_x_at0.00424T-cell receptor rearranged beta-chain V-region

213958_at0.00424CD6 antigen

220999_s_at0.00424synonyms: PIR121, PRO1331

205005_s_at0.00424N-myristoyltransferase 2

210707_x_at0.00487human PMS2 related gene

212414_s_at0.00487Septin 6

200965_s_at0.00487Actin binding LIM protein 1

211272_s_at0.00558Diacylglycerol kinase, alpha 80kDa

205255_x_at0.00558Transcription factor 7

203580_s_at0.00638Solute carrier family 7, member 6

218877_s_at0.00638Chromosome 6 open reading frame 75

216945_x_at0.00728PAS domain containing serine/threonine kinase

223162_s_at0.00728LCHN protein

203387_s_at0.00728TBC1 domain family, member 4

241871_at0.00728Calcium/calmodulin-dependent protein kinase IV

211339_s_at0.00728IL2-inducible T-cell kinase

227262_at0.00944Hyaluronan and proteoglycan link protein 3

213540_at0.00944

221558_s_at0.00944Lymphoid enhancer-binding factor 1

221712_s_at0.0107Hypothetical protein FLJ10439

211796_s_at0.0107T-cell receptor active beta-chain

39248_at0.0107Aquaporin 3

209504_s_at0.0122Pleckstrin homology domain containing, family B (evectins) member 1

201522_x_at0.0122SNRPN upstream reading frame

226272_at0.0138yx88c06.s1 Soares melanocyte 2NbHM Homo sapiens

205006_s_at0.0138N-myristoyltransferase 2

210948_s_at0.0138Lymphoid enhancer-binding factor 1

212096_s_at0.0138Mitochondrial tumor suppressor 1

228879_at0.0155602549172F1 NIH_MGC_61 Homo sapiens

205590_at0.0155RAS guanyl releasing protein 1

219765_at0.0175Hypothetical protein FLJ12586

204612_at0.0175Protein kinase inhibitor alpha

212413_at0.0175Septin 6

232001_at0.0186Hypothetical gene supported by AY007155

217729_s_at0.0197Amino-terminal enhancer of split

217950_at0.0197Nitric oxide synthase interacting protein

229264_at0.0197FLJ39739 protein

228109_at0.0222Ras protein-specific guanine nucleotide-releasing factor 2

216232_s_at0.0222GCN1 general control of amino-acid synthesis 1-like 1

227449_at0.0249EPH receptor A4

206042_x_at0.0249SNRPN upstream reading frame

219724_s_at0.0278

229029_at0.0311Transcribed locus, weakly similar to XP_499236.1 LOC442377

205254_x_at0.0311Transcription factor 7 (T-cell specific)

204777_s_at0.0347Mal, T-cell differentiation protein

213093_at0.0347Protein kinase C, alpha

238823_at0.0347Formin-like 3

227072_at.0387Rotatin

228960_at0.0387NMDA receptor regulated 2

218648_at0.0387Transducer of regulated cAMP response element-binding protein (CREB) 3

218510_x_at0.0408Hypothetical protein FLJ20152

224838_at0.043Forkhead box P1


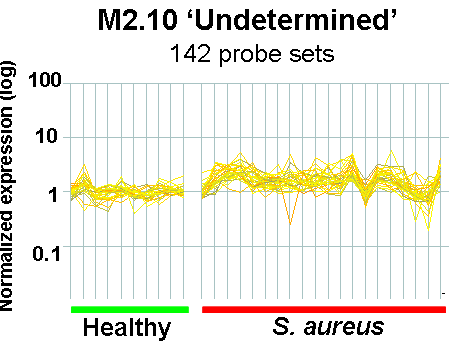


**Gene Probe** **p-Value** **Description**

208923_at0.00051Cytoplasmic FMR1 interacting protein 1

201944_at0.0006Hexosaminidase B (beta polypeptide)

203922_s_at0.000705Cytochrome b-245, beta polypeptide

211794_at0.000968FYN binding protein

219806_s_at0.00179FN5 protein

232617_at0.0024Cathepsin S

228766_at0.0024CD36 antigen

221841_s_at0.00278Kruppel-like factor 4 (gut)

226459_at0.00424Phosphoinositide-3-kinase adaptor protein 1

223922_x_at0.00424Membrane-spanning 4-domains, subfamily A, member 6A

238513_at0.00558Proline rich Gla (G-carboxyglutamic acid) 4

225171_at0.00638Rho GTPase activating protein 18

224702_at0.00638Hypothetical protein MGC23909

223498_at0.0083Sperm antigen HCMOGT-1

211366_x_at0.0083Caspase 1, apoptosis-related cysteine protease

224356_x_at0.00944Membrane-spanning 4-domains, subfamily A, member 6A

223204_at0.0138Hypothetical protein DKFZp434L142

209970_x_at0.0138Caspase 1, apoptosis-related cysteine protease 211367_s_at0.0155Caspase 1, apoptosis-related cysteine protease 223592_s_at0.0155Ring finger protein 135

221539_at0.0175Eukaryotic translation initiation factor 4E binding protein 1

226507_at0.0197P21/Cdc42/Rac1-activated kinase 1

226137_at0.0222High mobility group nucleosomal binding domain 3

223344_s_at0.0222Membrane-spanning 4-domains, subfamily A, member 7

201576_s_at0.0347Galactosidase, beta 1

229937_x_at0.0347Leukocyte immunoglobulin-like receptor, subfamily B

235054_at0.043Nudix -type motif 16

224983_at0.043Scavenger receptor class B, member 2

205726_at0.0477Diaphanous homolog 2


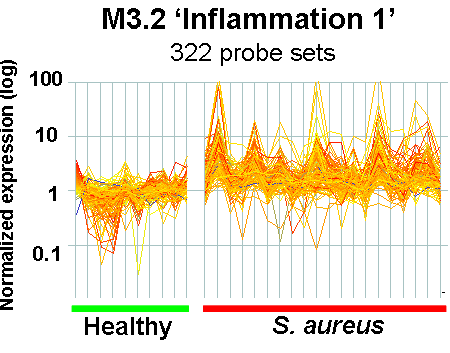


**Gene Probe** **p-Value** **Description**

228846_at1.62e-5MAX dimerization protein 1

203574_at2.92e-5Nuclear factor, interleukin 3 regulated

200663_at3.54e-5CD63 antigen (melanoma 1 antigen)

226275_at4.29e-5MAX dimerization protein 1

211924_s_at7.51e-5Plasminogen activator, urokinase receptor

214211_at7.51e-5Ferritin, heavy polypeptide 1

202859_x_at7.51e-5Interleukin 8

201858_s_at9.02e-5Proteoglycan 1, secretory granule

205349_at0.000108Guanine nucleotide binding protein (G protein), alpha 15

203140_at0.000108B-cell CLL/lymphoma 6 (zinc finger protein 51)

211506_s_at0.000108Homo sapiens IL 8 C-terminal variant (IL8) mRNA

217739_s_at0.000155Pre-B-cell colony enhancing factor 1

203887_s_at0.000155Thrombomodulin

225498_at0.000155Chromosome 20 open reading frame 178

220088_at0.000184Complement component 5 receptor 1 (C5a ligand)

215001_s_at0.000184Glutamate-ammonia ligase (glutamine synthase)

219681_s_at0.000219RAB11 family interacting protein 1 (class I)

203234_at0.000219Uridine phosphorylase 1

210512_s_at0.000219Vascular endothelial growth factor

224164_at0.000309Tropomyosin 3

203888_at0.000309Thrombomodulin

217202_s_at0.000366Homo sapiens glutamine synthetase pseudogene

235407_at0.000366602506825F1 NIH_MGC_79 Homo sapiens

228340_at0.000366601660648R1 NIH_MGC_72 Homo sapiens

202241_at0.000366Tribbles homolog 1 (Drosophila)

225142_at0.000432KIAA1718 protein

208937_s_at0.00051Inhibitor of DNA binding 1

201489_at0.00051Peptidylprolyl isomerase F (cyclophilin F)

225612_s_at0.000517d27a05.x1 NCI_CGAP_Pr28 Homo sapiens

243296_at0.0006Pre-B-cell colony enhancing factor 1

204908_s_at0.000705B-cell CLL/lymphoma 3

200919_at0.000705Polyhomeotic-like 2

228918_at0.000705Solute carrier family 43, member 2

210845_s_at0.000827Plasminogen activator, urokinase receptor

202284_s_at0.000968Cyclin-dependent kinase inhibitor 1A (p21, Cip1)

212769_at0.000968Transducin-like enhancer of split 3

217591_at0.00113SKI-like

224828_at0.00113ai42d03.s1 Soares_parathyroid_tumor_NbHPA

222088_s_at0.00113Solute carrier family 2, member 3

212723_at0.00132Phosphatidylserine receptor

217738_at0.00154Pre-B-cell colony enhancing factor 1

225884_s_at0.00154Zinc finger protein 336

202498_s_at0.00179Solute carrier family 2, member 3

216268_s_at0.00207Jagged 1

205767_at0.00207Epiregulin

205681_at0.00207BCL2-related protein A1

214696_at0.00207Homo sapiens clone 24659 mRNA sequence.

205409_at0.0024FOS-like antigen 2

227195_at0.0024Zinc finger protein 503

38037_at0.0024Heparin-binding EGF-like growth factor

203821_at0.0024Heparin-binding EGF-like growth factor

216260_at0.00278Dicer1, Dcr-1 homolog

205220_at0.00278G protein-coupled receptor 109B

240038_at0.00278Elongation factor, RNA polymerase II, 2

214108_at0.00278MAX protein

238719_at0.00278Protein phosphatase 2 (formerly 2A)

201531_at0.00278Zinc finger protein 36, C3H type, homolog

222670_s_at0.00278V-maf musculoaponeurotic fibrosarcoma oncogene

218880_at0.0032FOS-like antigen 2

225954_s_at0.00369Midnolin

200798_x_at0.00369Myeloid cell leukemia sequence 1

221654_s_at0.00424Ubiquitin specific protease 3

227060_at0.00424Tumor necrosis factor receptor superfamily, member 19-like

218559_s_at0.00424V-maf musculoaponeurotic fibrosarcoma oncogene homolog B

210542_s_at0.00424Solute carrier organic anion transporter family, member 3A1

216015_s_at0.00424Cold autoinflammatory syndrome 1

225582_at0.00487KIAA1754

224891_at0.00487Forkhead box O3A

207075_at0.00558Cold autoinflammatory syndrome 1

225557_at0.00558AXIN1 up-regulated 1

224692_at0.00558

203455_s_at0.00638Spermidine/spermine N1-acetyltransferase

230170_at0.00638Oncostatin M

201631_s_at0.00638Immediate early response 3

201329_s_at0.00638V-ets erythroblastosis virus E26 oncogene homolog 2

214721_x_at0.00638CDC42 effector protein 4

210592_s_at0.00728Spermidine/spermine N1-acetyltransferase

202672_s_at0.00728Activating transcription factor 3

212770_at0.00728Transducin-like enhancer of split 3

229221_at0.0083CD44 antigen

238893_at0.0083Hypothetical protein LOC338758

236224_at0.0083Transcribed locus

209099_x_at0.00885Jagged 1

200989_at0.00944Hypoxia-inducible factor 1, alpha subunit

224831_at0.0101ai42d03.s1 Soares_parathyroid_tumor_NbHPA Homo sapiens

201739_at0.0107Serum/glucocorticoid regulated kinase

225673_at0.0107601498152F1 NIH_MGC_70 Homo sapiens

37028_at0.0107Protein phosphatase 1, regulatory (inhibitor) subunit 15A

219434_at0.0107Triggering receptor expressed on myeloid cells 1

202071_at0.0107Syndecan 4 (amphiglycan, ryudocan)

231990_at0.0107Ubiquitin specific protease 15

201943_s_at0.0122Carboxypeptidase D

209803_s_at0.0122Pleckstrin homology-like domain, family A, member 2

225955_at0.0138Full length insert cDNA clone ZB42D04

209536_s_at0.0138Homo sapiens hepatocellular carcinoma-associated protein HCA10 mRNA, complete cds.

220740_s_at0.0138Solute carrier family 12 (potassium/chloride transporters), member 6

223454_at0.0155Chemokine (C-X-C motif) ligand 16

203411_s_at0.0155Lamin A/C

243931_at0.0175yi22f12.r1 Soares placenta Nb2HP Homo sapiens

202082_s_at0.0175SEC14-like 1 (S. cerevisiae)

239486_at0.0175Transcribed locus

239124_at0.0175Phosphatidylinositol transfer protein, alpha

206472_s_at0.0175Transducin-like enhancer of split 3 (E(sp1) homolog, Drosophila)

237502_at0.0197Chromosome 20 open reading frame 155

213524_s_at0.0197Putative lymphocyte G0/G1 switch gene

202637_s_at0.0197Intercellular adhesion molecule 1 (CD54), human rhinovirus receptor

224920_x_at0.0222Myeloid-associated differentiation marker

244025_at0.0222Transcribed locus

235242_at0.0222601556492F1 NIH_MGC_58 Homo sapiens

202464_s_at0.02226-phosphofructo-2-kinase/fructose-2,6-biphosphatase 3

201573_s_at0.0222Eukaryotic translation termination factor 1

212086_x_at0.0222Lamin A/C

210190_at0.0235Syntaxin 11

208092_s_at0.0249Family with sequence similarity 49, member A

202497_x_at0.0249Solute carrier family 2, member 3

235479_at0.0249Cytoplasmic polyadenylation element binding protein 2

201490_s_at0.0278Peptidylprolyl isomerase F

224836_at0.0278

202014_at0.0278Protein phosphatase 1, regulatory subunit 15A

202656_s_at0.0278SERTA domain containing 2

207674_at0.0278Fc fragment of IgA, receptor for

223186_at0.0311Ubiquitin-conjugating enzyme E2 variant 1

239494_at0.0311F-box and WD-40 domain protein 7

230492_s_at0.0311Hypothetical protein KIAA1434

208869_s_at0.0311GABA(A) receptor-associated protein like 1

227534_at0.0347Chromosome 9 open reading frame 21

225262_at0.0347LOC440853

224572_s_at0.0347Interferon regulatory factor 2 binding protein 2

201543_s_at0.0347SAR1a gene homolog 1 (S. cerevisiae)

239598_s_at0.0347Hypothetical protein FLJ20481

242176_at.0347aa47c07.s1 NCI_CGAP_GCB1 Homo sapiens

201324_at0.0347Epithelial membrane protein 1

219257_s_at0.0347Sphingosine kinase 1

212432_at0.0387GrpE-like 1, mitochondrial (E. coli)

64488_at0.0387CDNA FLJ38849 fis, clone MESAN2008936

217741_s_at0.0387Zinc finger, A20 domain containing 2

201170_s_at0.0387Basic helix-loop-helix domain containing, class B, 2

203471_s_at0.043Pleckstrin

204564_at0.043Polycomb group ring finger 3

212722_s_at0.043Phosphatidylserine receptor

209545_s_at0.0477Receptor-interacting serine-threonine kinase 2

222408_s_at0.0477Yippee-like 5 (Drosophila)

205020_s_at0.0477ADP-ribosylation factor-like 4A

201410_at0.0477Pleckstrin homology domain containing, family B member 2

212458_at0.0477Sprouty-related, EVH1 domain containing 2


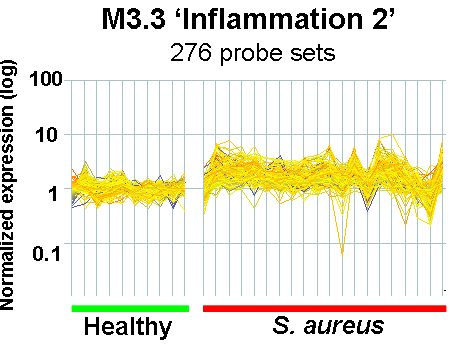


**Gene Probe** **p-Value** **Description**

203042_at9.02e-5Lysosomal-associated membrane protein 2

212268_at0.000108Serine (or cysteine) proteinase inhibitor, clade B member 1

226817_at0.000108Desmocollin 2

203126_at0.000155Inositol(myo)-1(or 4)-monophosphatase 2

211275_s_at0.000184Glycogenin

224918_x_at0.000184Microsomal glutathione S-transferase 1

201463_s_at0.000184Transaldolase 1

218450_at0.000219Heme binding protein 1

205147_x_at0.000219Neutrophil cytosolic factor 4, 40kDa

206130_s_at0.000219Asialoglycoprotein receptor 2

206488_s_at0.000261CD36 antigen

217995_at0.000261Sulfide quinone reductase-like (yeast)

205863_at0.000261S100 calcium binding protein A12 (calgranulin C)

217977_at0.000261Selenoprotein X, 1

200736_s_at0.000261Glutathione peroxidase 1

211571_s_at0.000309Chondroitin sulfate proteoglycan 2 (versican)

204620_s_at0.000366Chondroitin sulfate proteoglycan 2 (versican)

202990_at0.000366Phosphorylase, glycogen; liver

218660_at0.000366Dysferlin, limb girdle muscular dystrophy 2B

221210_s_at0.000366N-acetylneuraminate pyruvate lyase

231736_x_at0.000432Microsomal glutathione S-transferase 1

206584_at0.000432Lymphocyte antigen 96

201470_at0.000432Glutathione S-transferase omega 1

200975_at0.00051Palmitoyl-protein thioesterase 1

212807_s_at0.00051Sortilin 1

221523_s_at0.00051

213572_s_at0.00051Serine (or cysteine) proteinase inhibitor, clade B member 1

204214_s_at0.0006RAB32, member RAS oncogene family

204714_s_at0.0006Coagulation factor V

204249_s_at0.0006LIM domain only 2

205931_s_at0.000705CAMP responsive element binding protein 5

201118_at0.000705Phosphogluconate dehydrogenase

209154_at0.000705Tax1 binding protein 3

202671_s_at0.000827Pyridoxal kinase

210386_s_at0.000968Metaxin 1

202096_s_at0.000968Benzodiazapine receptor

212335_at0.00113Glucosamine (N-acetyl)-6-sulfatase

203175_at0.00113Ras homolog gene family, member G

222409_at0.00113Coronin, actin binding protein, 1C

215646_s_at0.00132Chondroitin sulfate proteoglycan 2

209005_at0.00132F-box and leucine-rich repeat protein 5

205174_s_at0.00132Glutaminyl-peptide cyclotransferase

208454_s_at0.00132Plasma glutamate carboxypeptidase

210427_x_at0.00132Annexin A2

235696_at0.00154Homo sapiens, clone IMAGE:4837650, mRNA, partial cds

215952_s_at0.00154Hypothetical protein FLJ14668

225059_at0.00179Angiotensin II receptor-associated protein

223158_s_at0.00179NIMA (never in mitosis gene a)-related kinase 6

214665_s_at0.00179Calcium binding protein P22

200999_s_at0.00207Cytoskeleton-associated protein 4

208074_s_at0.00207Adaptor-related protein complex 2, sigma 1 subunit

235678_at0.00207GM2 ganglioside activator

220990_s_at0.00207Likely ortholog of rat vacuole membrane protein 1

220034_at0.00207Interleukin-1 receptor-associated kinase 3

202377_at0.0024Leptin receptor

211742_s_at0.0024Ecotropic viral integration site 2B

222833_at0.0024Hypothetical protein FLJ20481

222688_at0.00278Phytoceramidase, alkaline

211047_x_at0.00278Adaptor-related protein complex 2, sigma 1 subunit

225129_at0.00278Copine II

202626_s_at0.00278V-yes-1 Yamaguchi sarcoma viral related oncogene

207168_s_at0.00278H2A histone family, member Y

235072_s_at0.0032Transcribed locus

234312_s_at0.0032Acetyl-Coenzyme A synthetase 2

218109_s_at0.0032Major facilitator superfamily domain containing 1

208926_at0.0032Sialidase 1

228083_at0.0032Calcium channel, voltage-dependent, alpha 2/delta subunit 4

203665_at0.0032Heme oxygenase (decycling) 1

204445_s_at0.00369Arachidonate 5-lipoxygenase

203184_at0.00369Fibrillin 2

238455_at0.00424Plexin domain containing 2

226865_at0.00424MRNA; cDNA DKFZp564O0862

201676_x_at0.00424Proteasome (prosome, macropain) subunit, alpha type, 1

222143_s_at0.00487Hypothetical protein FLJ22405

207467_x_at0.00487Calpastatin

208771_s_at0.00487Leukotriene A4 hydrolase

208700_s_at0.00487Transketolase

201238_s_at0.00487Capping protein (actin filament) muscle Z-line, alpha 2

209522_s_at0.00487Carnitine acetyltransferase

208736_at0.00558Actin related protein 2/3 complex, subunit 3, 21kDa

217947_at0.00558Chemokine-like factor super family 6

211746_x_at0.00558Proteasome (prosome, macropain) subunit, alpha type, 1

224796_at0.00558Development and differentiation enhancing factor 1

203912_s_at0.00638Deoxyribonuclease I-like 1

204099_at0.00638SWI/SNF related, actin dependent regulator of chromatin, subfamily d

218606_at0.00638Zinc finger, DHHC domain containing 7

215399_s_at0.00638tw54e04.x1 NCI_CGAP_Ut1 Homo sapiens

215706_x_at0.00728Zyxin

222447_at0.00728DORA reverse strand protein 1

210423_s_at0.00728Solute carrier family 11, member 1

218945_at0.00728Hypothetical protein MGC2654

229228_at0.00728CDNA FLJ32589 fis, clone SPLEN2000443

208699_x_at0.00728Transketolase

200602_at0.0083Amyloid beta (A4) precursor protein

214085_x_at0.0083HIV-1 rev binding protein 2

204168_at0.0083Microsomal glutathione S-transferase 2

205786_s_at0.0083Integrin, alpha M

201290_at0.0083SEC11-like 1

217868_s_at0.0083DORA reverse strand protein 1

222687_s_at0.0083Phytoceramidase, alkaline

203799_at0.00944Type I transmembrane C-type lectin receptor DCL-1

218404_at0.00944Sorting nexin 10

214629_x_at0.00944Reticulon 4

205844_at0.00944Vanin 1

205639_at0.0107Acyloxyacyl hydrolase

229635_at0.0107CDNA clone IMAGE:4800262, partial cds

219938_s_at0.0107Proline-serine-threonine phosphatase interacting protein 2

220001_at0.0107Peptidyl arginine deiminase, type IV

227069_at0.0107FP6778

231029_at0.0114Transcribed locus

212830_at0.0122EGF-like-domain, multiple 5

201379_s_at0.0122Tumor protein D52-like 2

204362_at0.0122Src family associated phosphoprotein 2

234985_at0.0122Hypothetical protein LOC143458

244313_at0.0122Transcribed locus

226849_at0.0122KIAA1608

218383_at0.0122Chromosome 14 open reading frame 94

239084_at0.0122Synaptosomal-associated protein, 29kDa

201972_at0.0138ATPase, H+ transporting, lysosomal 70kDa, V1 subunit A

218189_s_at0.0138N-acetylneuraminic acid synthase (sialic acid synthase)

219549_s_at0.0138Reticulon 3

226364_at0.0138Huntingtin interacting protein 1

208908_s_at0.0138Calpastatin

223120_at0.0138Fucosidase, alpha-L- 2, plasma

224912_at0.0138Tetratricopeptide repeat domain 7A

212636_at0.0138

201311_s_at0.0138SH3 domain binding glutamic acid-rich protein like

221156_x_at0.0146Phosphatidylinositol glycan, class B

200821_at0.0155Lysosomal-associated membrane protein 2

213241_at0.0155Plexin C1

202888_s_at0.0155Alanyl (membrane) aminopeptidase

202788_at0.0155Mitogen-activated protein kinase-activated protein kinase 3

201007_at0.0175Hydroxyacyl-Coenzyme A dehydrogenase

213397_x_at0.0197Angiogenin, ribonuclease, RNase A family, 5

210968_s_at0.0197Reticulon 4

209004_s_at0.0197F-box and leucine-rich repeat protein 5

210715_s_at0.0222Serine protease inhibitor, Kunitz type, 2

213733_at0.0249Myosin IF

238996_x_at0.0278Aldolase A, fructose-bisphosphate

221492_s_at0.0278APG3 autophagy 3-like (S. cerevisiae)

221666_s_at0.0278PYD and CARD domain containing

214500_at0.0278H2A histone family, member Y

200808_s_at0.0278Zyxin

206643_at0.0294Histidine ammonia-lyase

202185_at0.0311Procollagen-lysine, 2-oxoglutarate 5-dioxygenase 3

230435_at0.0311FLJ30851 protein

218072_at0.0347COMM domain containing 9

209393_s_at0.0347Eukaryotic translation initiation factor 4E member 2

201887_at0.0347Interleukin 13 receptor, alpha 1

225603_s_at0.0347Hypothetical protein LOC286144

205158_at0.0387Angiogenin, ribonuclease, RNase A family, 5

225372_at0.0387Full-length cDNA clone CS0DI015YJ11

209473_at0.0387Weakly similar to zinc finger protein 195

227776_at0.0387Transcribed locus, weakly similar to NP_775735.1

234978_at0.0387Solute carrier family 36, member 4

200827_at0.043Procollagen-lysine 1, 2-oxoglutarate 5-dioxygenase 1

201312_s_at0.043SH3 domain binding glutamic acid-rich protein like

224647_at0.0477Chromosome 10 open reading frame 9

204500_s_at0.0477ATP/GTP binding protein 1

202787_s_at0.0477Mitogen-activated protein kinase-activated protein kinase 3

213011_s_at0.0477Triosephosphate isomerase 1


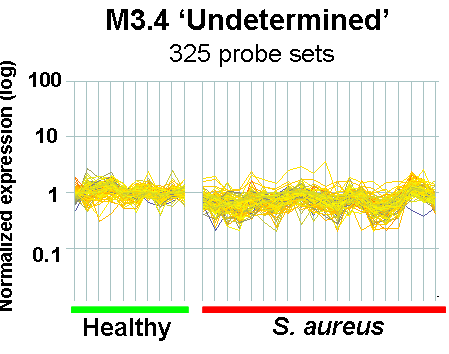


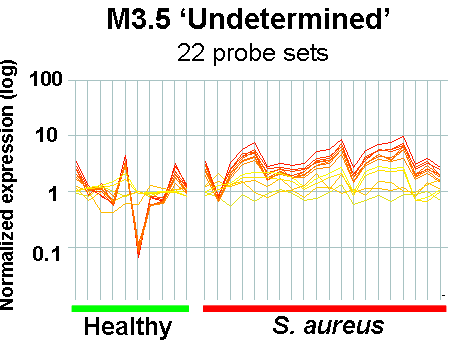


**Gene Probe** **p-Value** **Description**

214414_x_at0.0006Hemoglobin, alpha 1

209458_x_at0.000968Hemoglobin, alpha 1

217414_x_at0.00113Human alpha-globin gene with flanks.

211745_x_at0.00132Hemoglobin, alpha 2

209116_x_at0.0024Human sickle cell beta-globin mRNA

211696_x_at0.0024Hemoglobin, beta

204018_x_at0.0024Hemoglobin, alpha 1

217232_x_at0.00278Homo sapiens mutant beta-globin (HBB) gene

243576_at0.0175Transcribed locus

208755_x_at0.0249H3 histone, family 3B (H3.3B)

202264_s_at0.0347Translocase of outer mitochondrial membrane 40 homolog

208749_x_at0.0477Flotillin 1

213828_x_at0.0477H3 histone, family 3B (H3.3B)

**Gene Probe** **p-Value** **Description**

203378_at 0.000129 Pre-mRNA cleavage complex II protein Pcf11

37590_g_at 0.000184 Hypothetical protein DKFZp547K1113

210346_s_at 0.000184 CDC-like kinase 1

218263_s_at 0.000261 Transposon-derived Buster1 transposase-like protein

202832_at 0.000261 GRIP and coiled-coil domain containing 2

218499_at 0.000309 Mst3 and SOK1-related kinase

204651_at 0.00051 Nuclear respiratory factor 1

201934_at 0.000705 Hypothetical protein PRO2730

208127_s_at 0.000968 Suppressor of cytokine signaling 5

212847_at 0.000968 Nexilin \

221970_s_at 0.00113 DKFZP586L0724 protein

219378_at 0.00113 NMDA receptor regulated 1-like

221229_s_at 0.00113 Hypothetical protein FLJ20628

212267_at 0.00113 KIAA0261

212774_at 0.00113 Homo sapiens RP58 gene, complete CDS.

207606_s_at 0.00154 Rho GTPase activating protein 12

221510_s_at 0.00154 Glutaminase

214323_s_at 0.00154 UPF3 regulator of nonsense transcripts homolog A

218649_x_at 0.00179 Serologically defined colon cancer antigen 1

204593_s_at 0.00179 Hypothetical protein FLJ20232

213025_at 0.00207 DKFZp762M0710_s1 762 (synonym: hmel2) 207483_s_at 0.00207 TBP-interacting protein

218432_at 0.00223 F-box protein 3

212366_at 0.0024 Zinc finger protein 292

221020_s_at 0.00278 Mitochondrial folate transporter/carrier

212455_at 0.00369 Splicing factor YT521-B

214855_s_at 0.00369 GTPase activating Rap/RanGAP domain-like 1

220553_s_at 0.00369

202880_s_at 0.00369 Pleckstrin homology, Sec7 and coiled-coil domains

202265_at 0.00487 Polycomb group ring finger 4

221826_at 0.00558 Similar to RIKEN cDNA 2610307I21

203356_at 0.00558 Calpain 7

212180_at 0.00558 V-crk sarcoma virus CT10 oncogene homolog (avian)-like

208884_s_at 0.00558 E3 identified by differential display

217987_at 0.00558 HCV NS3-transactivated protein 1

206695_x_at 0.00558 Zinc finger protein 43 (HTF6)

201486_at 0.00558 Reticulocalbin 2, EF-hand calcium binding domain

213049_at 0.00558 GTPase activating Rap/RanGAP domain-like 1

203301_s_at 0.00638 Cyclin D binding myb-like transcription factor 1

204847_at 0.00638 Zinc finger and BTB domain containing 11

202853_s_at 0.00638 RYK receptor-like tyrosine kinase

202703_at 0.00638 Dual specificity phosphatase 11

203525_s_at 0.00638 Adenomatosis polyposis coli

203544_s_at 0.00638 Signal transducing adaptor molecule

202097_at 0.00638 Nucleoporin 153kDa

218067_s_at 0.00638 Hypothetical protein FLJ10154

209724_s_at 0.00638 Zinc finger protein 161 homolog

212231_at 0.00728 F-box protein 21

219031_s_at 0.00728 Comparative gene identification transcript 37

221596_s_at 0.00728 Hypothetical protein DKFZp564O0523

212418_at 0.0083 E74-like factor 1

212058_at 0.0083 U2-associated SR140 protein

212239_at 0.0083 Phosphoinositide-3-kinase, regulatory subunit 1

217833_at 0.0083 Synaptotagmin binding

201314_at 0.00944 Serine/threonine kinase 25 (STE20 homolog, yeast)

218878_s_at 0.00944 Sirtuin

212787_at 0.00944 YLP motif containing 1

203250_at 0.00944 RNA binding motif protein 16

213070_at 0.00944 Phosphoinositide-3-kinase, class 2, alpha polypeptide

212855_at 0.00944 KIAA0276 protein

203852_s_at 0.00944 Survival of motor neuron 1, telomeric

217828_at 0.0107 Hypothetical protein FLJ13213

200686_s_at 0.0107 Splicing factor, arginine/serine-rich 11

206015_s_at 0.0107 Forkhead box J3

221257_x_at 0.0107 F-box protein 38

203620_s_at 0.0122 FCH and double SH3 domains 2

208896_at 0.0122 DEAD (Asp-Glu-Ala-Asp) box polypeptide 18

215245_x_at 0.0122 Fragile X mental retardation 1

211185_s_at 0.0122 Hypothetical protein FLJ14753

209025_s_at 0.0122 Synaptotagmin binding, cytoplasmic RNA interacting protein

212615_at 0.0122 Chromodomain helicase DNA binding protein 9

207956_x_at 0.0138 Androgen-induced proliferation inhibitor

201260_s_at 0.0138 Synaptophysin-like protein

205596_s_at 0.0155 SMAD specific E3 ubiquitin protein ligase 2

212920_at 0.0155 Transcribed locus, weakly similar to XP_514612.1]

212842_x_at 0.0155 RAN binding protein 2-like 1

221230_s_at 0.0155 AT rich interactive domain 4B (RBP1- like)

208761_s_at 0.0155 SMT3 suppressor of mif two 3 homolog 1

202693_s_at 0.0155 Serine/threonine kinase 17a

203531_at 0.0155 Cullin 5

209741_x_at 0.0155 Zinc finger protein 291

218082_s_at 0.0175 Upstream binding protein 1

209022_at 0.0175 Stromal antigen 2

202541_at 0.0175 Small inducible cytokine subfamily E, member 1

212633_at 0.0175 UI-H-BW0-ajs-b-03-0-UI.s1 NCI_CGAP_Sub6 Homo sapiens

200899_s_at 0.0175 Meningioma expressed antigen 5

218247_s_at 0.0175 Ring finger and KH domain containing 2

212493_s_at 0.0175 Huntingtin interacting protein B

201865_x_at 0.0175 Nuclear receptor subfamily 3, group C, member 1

218149_s_at 0.0197 Homo sapiens zinc finger protein 395 (ZNF395), mRNA.

213111_at 0.0197 Phosphatidylinositol-3-phosphate/phosphatidylinositol 5-kinase, type III

209064_x_at 0.0197 Poly(A) binding protein interacting protein 1

204520_x_at 0.0197 Bromodomain containing 1

218352_at 0.0222 Regulator of chromosome condensation (RCC1) and BTB (POZ) domain containing protein 1

218696_at 0.0222 Eukaryotic translation initiation factor 2-alpha kinase 3

216321_s_at 0.0222 Nuclear receptor subfamily 3, group C, member 1

218079_s_at 0.0222 Zinc finger protein 403

209455_at 0.0249 F-box and WD-40 domain protein 11

203624_at 0.0249 DNA segment on chromosome X and Y 155 expressed sequence

218386_x_at 0.0249 Ubiquitin specific protease 16

219231_at 0.0249 Nuclear receptor coactivator 6 interacting protein

214835_s_at 0.0278 Succinate-CoA ligase, GDP-forming, beta subunit

201829_at 0.0278 Neuroepithelial cell transforming gene 1

218381_s_at 0.0311 U2 (RNU2) small nuclear RNA auxiliary factor 2

221559_s_at 0.0311 MIS12 homolog (yeast)

212990_at 0.0311 Synaptojanin 1

212764_at 0.0311 Human two-handed zinc finger protein ZEB mRNA, partial cds.

205061_s_at 0.0347 Exosome component 9

212752_at 0.0387 Cytoplasmic linker associated protein 1

218096_at 0.0387 1-acylglycerol-3-phosphate O-acyltransferase 5

217731_s_at 0.0387 Integral membrane protein 2B

202396_at 0.0387 Transcription elongation regulator 1

202034_x_at 0.0387 RB1-inducible coiled-coil 1

218236_s_at 0.0387 Protein kinase D3

217142_at 0.0387

209674_at 0.0387 Cryptochrome 1 (photolyase-like)

212579_at 0.043 KIAA0650 protein

39582_at 0.043 Cylindromatosis (turban tumor syndrome)

201031_s_at 0.0477 Heterogeneous nuclear ribonucleoprotein H1 (H)

209451_at 0.0477 TRAF family member-associated NFKB activator

209704_at 0.0477 Likely ortholog of mouse metal response element binding transcription factor 2

218566_s_at 0.0477 Cysteine and histidine-rich domain (CHORD)-containing, zinc binding protein 1

**Gene Probe** **p-Value** **Description**

220661_s_at1.09e-5Hypothetical protein FLJ20531

236165_at2.41e-5Male-specific lethal 3-like 1

218505_at2.92e-5FP977

225724_at3.54e-5Hypothetical protein FLJ31306

225017_at3.54e-5Hypothetical protein FLJ12892

219698_s_at4.29e-5Methyltransferase like 4

213670_x_at5.18e-5Williams-Beuren Syndrome critical region protein 20 copy B

202220_at6.24e-5KIAA0907 protein

226344_at6.24e-5Zinc finger, matrin type 1

214100_x_at6.24e-5Williams-Beuren Syndrome critical region protein 20 copy B

204352_at7.51e-5TNF receptor-associated factor 5

209271_at7.51e-5PHD finger transcription factor

230444_at7.51e-5Transcribed locus, moderately similar to XP_510104.1

202548_s_at9.02e-5Rho guanine nucleotide exchange factor (GEF) 7

208206_s_at9.02e-5RAS guanyl releasing protein 2

213460_x_at9.02e-5Williams Beuren syndrome chromosome region 20C

213653_at0.000108Methyltransferase like 3

203288_at0.000129KIAA0355

203384_s_at0.000129Golgi autoantigen, golgin subfamily a, 1

204552_at0.000129EST63624 Jurkat T-cells V Homo sapiens

227485_at0.000129DEAD/H (Asp-Glu-Ala-Asp/His) box polypeptide 26B

203804_s_at0.000155Cisplatin resistance-associated overexpressed protein

209007_s_at0.000155NPD014 protein

212277_at0.000184Myotubularin related protein 4

201394_s_at0.000184RNA binding motif protein 5

223716_s_at0.000184Zinc finger protein 265

217122_s_at0.000184

203551_s_at0.000184COX11 homolog, cytochrome c oxidase assembly protein

215493_x_at0.000219

218373_at0.000219Fused toes homolog (mouse)

212454_x_at0.000261Heterogeneous nuclear ribonucleoprotein D-like

212914_at0.000261Chromobox homolog 7

32259_at0.000261Enhancer of zeste homolog 1 (Drosophila)

221971_x_at0.000261Similar to centaurin, gamma-like family, member 1

214093_s_at0.000309Far upstream element (FUSE) binding protein 1

206828_at0.000309TXK tyrosine kinase

236832_at0.000309Hypothetical protein LOC221442

218921_at0.000309Single Ig IL-1R-related molecule

231940_at0.000366Zinc finger protein 529

228023_x_at0.000366AV699389 GKC Homo sapiens cDNA

203249_at0.000366Enhancer of zeste homolog 1 (Drosophila)

204706_at0.000432Inositol polyphosphate-5-phosphatase, 72 kDa

219447_s_at0.000432Solute carrier family 35, member C2

227489_at0.000432601655271R1 NIH_MGC_65 Homo sapiens

212944_at0.000432Mitochondrial ribosomal protein S6

218366_x_at0.000432FLJ20859 gene

213773_x_at0.000432NOL1/NOP2/Sun domain family, member 5

203014_x_at0.00051RUN and TBC1 domain containing 3

52940_at0.00051Single Ig IL-1R-related molecule

213398_s_at0.00051Chromosome 14 open reading frame 124

213474_at0.00051Potassium channel tetramerisation domain containing 7

227751_at0.00051Programmed cell death 5

203579_s_at0.00051Solute carrier family 7, member 6

203802_x_at0.000553NOL1/NOP2/Sun domain family, member 5

213140_s_at0.0006Synovial sarcoma translocation gene on chromosome 18

215743_at0.0006Ribonuclease P/MRP 38kDa subunit

225786_at0.0006Family with sequence similarity 36, member A

236079_at0.000705Transcribed locus

219870_at0.000705Activating transcription factor 7 interacting protein 2

232171_x_at0.000705Kelch domain containing 4

219095_at0.000705Phospholipase A2, group IVB (cytosolic)

242607_at0.000705Transcribed locus

241991_at0.000705Hypothetical protein BC017868

214686_at0.000827MRNA; cDNA DKFZp686N1450

212232_at0.000827Formin binding protein 4

208634_s_at0.000827Microtubule-actin crosslinking factor 1

225839_at0.000827Hypothetical protein LOC155435

218456_at0.000827C1q domain containing 1

217940_s_at0.000827Hypothetical protein FLJ10769

218315_s_at0.000827CDK5 regulatory subunit associated protein 1

229366_at0.000827Cereblon

217895_at0.000827FLJ20758 protein

219627_at0.000827Hypothetical protein FLJ12700

235484_at0.000968601435734F1 NIH_MGC_72 Homo sapiens

202979_s_at0.000968HCF-binding transcription factor Zhangfei

221264_s_at0.000968

213587_s_at0.00113Hypothetical gene LOC401431

228282_at0.00113Hypothetical protein MGC33302

228157_at0.00113Zinc finger protein 207

218873_at0.00113FLJ12923

226015_at0.00113Zinc finger protein 12

213213_at0.00113

236436_at0.00132Hypothetical protein LOC283130

203944_x_at0.00132Butyrophilin, subfamily 2, member A1

208798_x_at0.00132Golgin-67

228334_x_at0.00132KIAA1712

227603_at0.00132GLI-Kruppel family member HKR1

224817_at0.00132SH3 multiple domains 1

206150_at0.00154Tumor necrosis factor receptor superfamily, member 7

218552_at0.00154Enoyl Coenzyme A hydratase domain containing 2

213278_at0.00154Myotubularin related protein 9

228216_at0.00154wx71b04.x1 NCI_CGAP_Brn53 Homo sapiens e.

203117_s_at0.00154Ubiquitin specific protease 52

204773_at0.00179Interleukin 11 receptor, alpha

228736_at0.00179DNA helicase HEL308

221740_x_at0.00179602319218F1 NIH_MGC_89 Homo sapiens

215307_at0.00179MRNA full length insert cDNA clone EUROIMAGE 31619

221626_at0.00207Zinc finger protein 506

218734_at0.00207Hypothetical protein FLJ13848

231850_x_at0.00207KIAA1712

221517_s_at0.00207Cofactor required for Sp1 transcriptional activation, subunit 6, 77kDa

218016_s_at0.0024Polymerase (RNA) III (DNA directed) polypeptide E (80kD)

228512_at0.0024FLJ20758 protein

220143_x_at0.0024LUC7-like (S. cerevisiae)

213326_at0.0024Vesicle-associated membrane protein 1 (synaptobrevin 1)

204294_at0.0024Aminomethyltransferase (glycine cleavage system protein T)

218370_s_at0.0024Hypothetical protein FLJ12903

211383_s_at0.00278WD repeat domain 37

228465_at0.00278Homo sapiens, clone IMAGE:5175565, mRNA

225112_at0.00278Abl interactor 2

202561_at0.00278Tankyrase

212179_at0.00278Chromosome 6 open reading frame 111

222147_s_at0.00278

214163_at0.00278Chromosome 1 open reading frame 41

228001_at0.00278Chromosome 21 open reading frame 4

203164_at0.0032Solute carrier family 33 (acetyl-CoA transporter), member 1

228318_s_at0.0032Hypothetical protein FLJ34443

213743_at0.0032Cyclin T2

229949_at0.0032General transcription factor II, i, pseudogene 1

226848_at0.0032Nuclear receptor subfamily 2, group C, member 2

220132_s_at0.00369Lectin-like NK cell receptor

219169_s_at0.00369Transcription factor B1, mitochondrial

221564_at0.00369HMT1 hnRNP methyltransferase-like 1

203578_s_at0.00369Solute carrier family 7, member 6

227504_s_at0.00424yz86g08.s1 Soares_multiple_sclerosis_2NbHMSP Homo sapiens

226635_at0.00424602322848F1 NIH_MGC_89 Homo sapiens

226316_at0.00487Chromosome 13 open reading frame 10

214473_x_at0.00558Postmeiotic segregation increased 2-like 3

227754_at0.00558CDNA FLJ10417 fis, clone NT2RP1000112

231890_at0.00558CDNA FLJ12742 fis, clone NT2RP2000644

229700_at0.00558601660507R1 NIH_MGC_71 Homo sapiens

220081_x_at0.00558Hydroxysteroid (17-beta) dehydrogenase 7

231944_at0.00597ERO1-like beta (S. cerevisiae)

207100_s_at0.00638Vesicle-associated membrane protein 1

205047_s_at0.00638Asparagine synthetase

202515_at0.00638DKFZP586B0319 protein

212176_at0.00638Chromosome 6 open reading frame 111

215954_s_at0.00638Chromosome 19 open reading frame 29

221965_at0.00638M-phase phosphoprotein 9

213186_at0.00638Zinc finger DAZ interacting protein 3

204143_s_at0.00728Enolase superfamily member 1

212037_at0.00728Pinin, desmosome associated protein

212851_at0.00728KIAA0276 protein

219147_s_at0.0083Chromosome 9 open reading frame 95

226334_s_at0.0083AHA1, activator of heat shock 90kDa protein ATPase homolog 2

225107_at0.0083LOC442518

209770_at0.0083Butyrophilin, subfamily 3, member A1

202104_s_at0.00944Spastic paraplegia 7, paraplegin

221080_s_at0.00944Family with sequence similarity 31, member C

225504_at0.00944Hypothetical protein FLJ21616

225760_at0.00944KIAA1915 protein

40569_at0.00944Human zinc finger protein 42 (MZF-1) mRNA, complete cds.

202789_at0.00944

209240_at0.00944O-linked N-acetylglucosamine (GlcNAc) transferase

218515_at0.00944Chromosome 21 open reading frame 66

220642_x_at0.0107G protein-coupled receptor 89

220035_at0.0107Nucleoporin 210kDa

220418_at0.0107Ubiquitin associated and SH3 domain containing, A

213672_at0.0107Methionine-tRNA synthetase

219151_s_at0.0107RAB, member of RAS oncogene family-like 2B

229384_at0.0122CDNA FLJ42101 fis, clone TESOP2006704

204538_x_at0.0122Nuclear pore complex interacting protein

218343_s_at0.0122General transcription factor IIIC, polypeptide 3, 102kDa

212819_at0.0138Ankyrin repeat and SOCS box-containing 1

239734_at0.0138Transcribed locus, weakly similar to XP_517454.1

214717_at0.0138Hypothetical protein DKFZp434H1419

226934_at0.0138Cleavage and polyadenylation specific factor 6, 68kDa

219648_at0.0155Whn-dependent transcript 2

227585_at0.0155ATPase family, AAA domain containing 1

230408_at0.0155Polycomb group ring finger 3

238620_at0.0155Transcribed locus

208718_at0.0155

220349_s_at0.0175Endo-beta-N-acetylglucosaminidase

226528_at0.0175Metaxin 3

209782_s_at0.0175D site of albumin promoter (albumin D-box) binding protein

222999_s_at0.0175Cyclin L2

228397_at0.0175Hypothetical protein FLJ20618

218476_at0.0197Protein-O-mannosyltransferase 1

227227_at0.0197Hypothetical LOC9884

43977_at0.0197Hypothetical protein FLJ20422

221834_at0.0197Seven in absentia homolog 1 (Drosophila)

221833_at0.0197Seven in absentia homolog 1 (Drosophila)

220607_x_at0.0197TH1-like (Drosophila)

220742_s_at0.0197N-glycanase 1

241863_x_at0.0222zj11c05.s1 Soares_fetal_liver_spleen_1NFLS_S1 Homo sapiens

204828_at0.0222RAD9 homolog A (S. pombe)

204791_at0.0222Nuclear receptor subfamily 2, group C, member 1

229078_s_at0.0222KIAA1704

219038_at0.0249Zinc finger, CW type with coiled-coil domain 2

228370_at0.02497i87g09.x1 NCI_CGAP_Ov18 Homo sapiens

226040_at0.0249MRNA; cDNA DKFZp762N156

224876_at0.0249Hypothetical protein FLJ37562

204060_s_at0.0249Protein kinase, X-linked

212753_at0.0278Polycomb group ring finger 3

243729_at0.0278CDNA FLJ37931 fis, clone CTONG2004397

205584_at0.0278Chromosome X open reading frame 45

207358_x_at0.0311Microtubule-actin crosslinking factor 1

226474_at0.0311Nucleotide-binding oligomerization domains 27

202098_s_at0.0311HMT1 hnRNP methyltransferase-like 1 (S. cerevisiae)

243362_s_at0.0311CDNA clone IMAGE:5288879, partial cds

229425_at0.0311zd61g09.s1 Soares_fetal_heart_NbHH19W Homo sapiens

215358_x_at0.0311Zinc finger protein 37b (KOX 21)

228331_at0.0311Chromosome 11 open reading frame 31

205760_s_at0.03298-oxoguanine DNA glycosylase

212557_at0.0329KIAA1702 protein

213882_at0.0347Beta-amyloid binding protein precursor

214048_at0.0347Methyl-CpG binding domain protein 4

201837_s_at0.0347SPTF-associated factor 65 gamma

203082_at0.0347BMS1-like, ribosome assembly protein (yeast)

231838_at0.0387Chromosome 20 open reading frame 119

213065_at0.0387Hypothetical protein MGC23401

221645_s_at0.0387Zinc finger protein 83 (HPF1)

212201_at0.0387KIAA0692 protein

213364_s_at0.0387Sorting nexin 1

203135_at0.0387TATA box binding protein

226995_at0.043AV705934 ADB Homo sapiens

235432_at0.043Nephronophthisis 3 (adolescent)

215667_x_at0.043Postmeiotic segregation increased 2-like 1

212126_at0.043Chromobox homolog 5

203707_at0.0477Zinc finger protein 263

212980_at0.0477Ubiquitin specific protease 34

221293_s_at0.0477Differentially expressed in FDCP 6 homolog

242273_at0.0477Transcribed locus


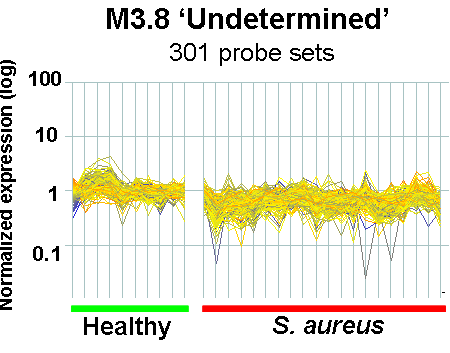

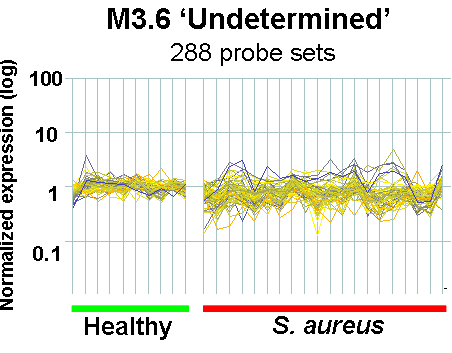


**Gene Probe** **p-Value** **Description**

231093_at9.02e-5Fc receptor-like protein 3

204683_at0.000108Intercellular adhesion molecule 2

228841_at0.000309CDNA FLJ32429 fis, clone SKMUS2001014

201756_at0.0006Replication protein A2, 32kDa

227293_at0.000968FIP1 like 1

231921_at0.00132Hypothetical protein FLJ13096

230298_at0.00207Similar to metallo-beta-lactamase superfamily protein

201491_at0.00207AHA1, activator of heat shock 90kDa protein ATPase homolog 1

226421_at0.0024Hypothetical protein LOC286505

227520_at0.00278Chromosome X open reading frame 15

225769_at0.00278Component of oligomeric golgi complex 6

236539_at0.0032Transcribed locus

228026_at0.0032Hypothetical protein FLJ21168

226116_at0.0032CDNA FLJ12540 fis, clone NT2RM4000425

235125_x_at0.0032Transcribed locus

223301_s_at0.00369Hypothetical protein FLJ23518

225502_at0.00369

222715_s_at0.00369AP1 gamma subunit binding protein 1

225512_at0.00369Hypothetical protein FLJ35036

227422_at0.00424Transcribed locus

222473_s_at0.00424Erbb2 interacting protein

226831_at0.00558Transcribed locus

225367_at0.00558Phosphoglucomutase 2

203008_x_at0.00638Thioredoxin domain containing 9

230003_at0.00638Transcribed locus

201742_x_at0.00944Splicing factor, arginine/serine-rich 1

226861_at0.00944Ankyrin repeat and SOCS box-containing 8

213698_at0.00944Zinc finger protein 258

226511_at0.0107AU157441 PLACE1 Homo sapiens

224974_at0.0107Likely ortholog of mouse Sds3

211337_s_at0.0107Gamma tubulin ring complex protein (76p gene)

219283_at0.0107COSMC

226019_at0.0138OMA1 homolog, zinc metallopeptidase

209600_s_at0.0138Acyl-Coenzyme A oxidase 1, palmitoyl

228495_at0.0146at05e07.x1 Barstead aorta HPLRB6 Homo sapiens cDNA clone 224919_at0.0155Mitochondrial ribosomal protein S6

224600_at0.0155CGG triplet repeat binding protein 1

226432_at0.0155CDNA clone IMAGE:5261903, partial cds

201716_at0.0175Sorting nexin 1

228283_at0.0197Hypothetical protein MGC61571

232024_at0.0197GTPase, IMAP family member 2

200617_at0.0197KIAA0152

229295_at0.0222Hypothetical protein LOC150166

223576_at0.0222Chromosome 6 open reading frame 203

226119_at0.0222Similar to hypothetical protein FLJ10883

227247_at0.0249Pleckstrin homology domain containing, family A member 8

228075_x_at0.0249Transcription factor B1, mitochondrial

224944_at0.0249AL566034 Homo sapiens FETAL BRAIN Homo sapiens cDNA.

225554_s_at0.0278Anaphase promoting complex subunit 7

222589_at0.0278Nemo like kinase

201634_s_at0.0311Hypothetical protein LOC283852

226628_at0.0311THO complex 2

225509_at0.0311Sin3A associated protein p30-like

235424_at0.0347Chromosome 9 open reading frame 42

223223_at0.0387ARV1 homolog

225366_at0.0387Phosphoglucomutase 2

208899_x_at 0.0387 ATPase, H+ transporting, lysosomal 34kDa, V1 subunit D

228987_at0.043Family with sequence similarity 49, member B

223104_at0.043Jagunal homolog 1

225439_at0.043Chronic myelogenous leukemia tumor antigen 66

225501_at0.0477Homo sapiens cDNA: FLJ23386 fis, clone HEP16928.

201746_at0.0477Tumor protein p53 (Li-Fraumeni syndrome)

223335_at0.0477Hypothetical protein LOC51249

222702_x_at0.0477Postsynaptic protein CRIPT

229804_x_at0.0477COBW domain containing 1

213149_at0.0477xs53f02.x1 NCI_CGAP_Kid11 Homo sapiens cDNA


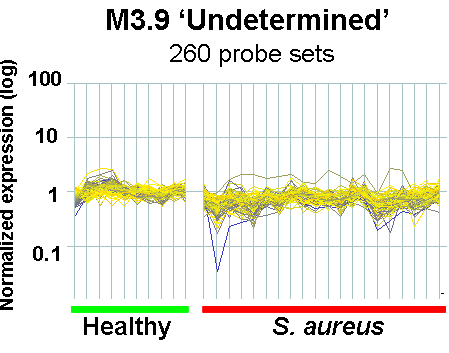


**Gene Probe** **p-Value** **Description**

222369_at 0.000184 Hypothetical protein FLJ13848

222201_s_at 0.000261 CASP8 associated protein 2

206542_s_at 0.000309 SWI/SNF related, actin dependent regulator of chromatin

218322_s_at 0.000309 Acyl-CoA synthetase long-chain family member 5

209092_s_at 0.000366 Chromosome 17 open reading frame 25

213090_s_at 0.000432 TAF4 RNA polymerase II

203537_at 0.0006 Phosphoribosyl pyrophosphate synthetase-associated protein 2

203513_at 0.000968 Hypothetical protein FLJ21439

201448_at 0.00113 TIA1 cytotoxic granule-associated RNA binding protein

219972_s_at 0.00113 Chromosome 14 open reading frame 135

218838_s_at 0.00132 Hypothetical protein FLJ12788

218699_at 0.00154 RAB7, member RAS oncogene family-like 1

227987_at 0.00179 Vacuolar protein sorting 13A (yeast)

38892_at 0.00179 KIAA0240

201967_at 0.0024 RNA binding motif protein 6

208861_s_at 0.00278 Alpha thalassemia/mental retardation syndrome X-linked

202491_s_at 0.0032 Inhibitor of kappa light polypeptide gene enhancer in B-cells

218701_at 0.0032 Lactamase, beta 2

212652_s_at 0.0032 Sorting nexin 4

201493_s_at 0.00369 Vacuolar protein sorting 35 (yeast)

218962_s_at 0.00455 Hypothetical protein FLJ13576

208070_s_at 0.00487 REV3-like, catalytic subunit of DNA polymerase zeta (yeast)

202602_s_at 0.00487 HIV TAT specific factor 1

208809_s_at 0.00487 Chromosome 6 open reading frame 62

201778_s_at 0.00487 KIAA0494 gene product

65472_at 0.00487 qb80a04.x1 Soares_fetal_heart_NbHH19W Homo sapiens

206308_at 0.00558 DNA (cytosine-5-)-methyltransferase 2

203791_at 0.00558 Dmx-like 1

218212_s_at 0.00638 Molybdenum cofactor synthesis 2

212486_s_at 0.00638 FYN oncogene related to SRC, FGR, YES

217970_s_at 0.00638 Carbon catabolite repression 4 protein

212754_s_at 0.0083 KIAA1040 protein

218842_at 0.0083 Hypothetical protein FLJ21908

221873_at 0.0083 Zinc finger protein 143 (clone pHZ-1)

217815_at 0.00944 Suppressor of Ty 16 homolog (S. cerevisiae)

212904_at 0.0107 KIAA1185 protein

204521_at 0.0107 Protein predicted by clone 23733

203688_at 0.0107 Polycystic kidney disease 2

203689_s_at 0.0107 Fragile X mental retardation 1

205668_at 0.0122 Lymphocyte antigen 75

212780_at 0.0122 Son of sevenless homolog 1

219363_s_at 0.0122 CGI-12 protein

201734_at 0.0138 Chloride channel 3

211954_s_at 0.0155 RAN binding protein 5

201166_s_at 0.0155 PRO0611 protein

204020_at 0.0155 Purine-rich element binding protein A

203741_s_at 0.0175 Adenylate cyclase 7

204333_s_at 0.0175 Aspartylglucosaminidase

212140_at 0.0175 SCC-112 protein

212499_s_at 0.0175 Chromosome 14 open reading frame 32

205771_s_at 0.0197

226685_at 0.0197 Syntrophin, beta 2

212513_s_at 0.0197 Ubiquitin specific protease 33

218593_at 0.0222 RNA binding motif protein 28

204849_at 0.0222 Transcription factor-like 5 (basic helix-loop-helix)

212299_at 0.0235 NIMA (never in mitosis gene a)- related kinase 9

214789_x_at 0.0249 Splicing factor, arginine/serine-rich, 46kD

203415_at 0.0249 Programmed cell death 6

202663_at 0.0249 Wiskott-Aldrich syndrome protein interacting protein

219137_s_at 0.0278 Chromosome 2 open reading frame 33

201517_at 0.0278 Nuclear cap binding protein subunit 2, 20kDa

213313_at 0.0278 RAB GTPase activating protein 1

219296_at 0.0278 Zinc finger, DHHC domain containing 13

38290_at 0.0311 Regulator of G-protein signalling 14

201034_at 0.0311 Chromosome 19 open reading frame 7

218396_at 0.0311 Vacuolar protein sorting 13C (yeast)

229342_at 0.0311 CDNA FLJ32162 fis, clone PLACE6000325

228548_at 0.0347 RAP1A, member of RAS oncogene family

219007_at 0.0347 Nucleoporin 43kDa

205052_at 0.0347 AU RNA binding protein/enoyl-Coenzyme A hydratase

200877_at 0.0347 Chaperonin containing TCP1, subunit 4

203738_at 0.0347 Hypothetical protein FLJ11193

210111_s_at 0.0387 KIAA0265 protein

218047_at 0.0387 Oxysterol binding protein-like 9

202261_at 0.0387 Transcription factor-like 1

203067_at 0.043 Pyruvate dehydrogenase complex, component X

212877_at 0.043 Kinesin 2 60/70kDa

203845_at 0.043 P300/CBP-associated factor

201817_at 0.043 Ubiquitin protein ligase E3C

219481_at 0.043 Tetratricopeptide repeat domain 13

208882_s_at 0.0477 E3 identified by differential display

201528_at 0.0477 Replication protein A1, 70kDa
